# Reassortment patterns of avian influenza virus internal segments among different subtypes

**DOI:** 10.1186/1471-2148-14-16

**Published:** 2014-01-24

**Authors:** Lu Lu, Samantha J Lycett, Andrew J Leigh Brown

**Affiliations:** 1 University of Edinburgh, Institute of Evolutionary Biology, Ashworth Laboratories, West Mains Road, Edinburgh EH9 3JT, UK; 2 Current address: University of Glasgow, Institute of Biodiversity, Animal Health and Comparative Medicine, Glasgow G12 8QQ, UK

## Abstract

**Background:**

The segmented RNA genome of avian Influenza viruses (AIV) allows genetic reassortment between co-infecting viruses, providing an evolutionary pathway to generate genetic innovation. The genetic diversity (16 haemagglutinin and 9 neuraminidase subtypes) of AIV indicates an extensive reservoir of influenza viruses exists in bird populations, but how frequently subtypes reassort with each other is still unknown. Here we quantify the reassortment patterns among subtypes in the Eurasian avian viral pool by reconstructing the ancestral states of the subtypes as discrete states on time-scaled phylogenies with respect to the internal protein coding segments. We further analyzed how host species, the inferred evolutionary rates and the d_N_/d_S_ ratio varied among segments and between discrete subtypes, and whether these factors may be associated with inter-subtype reassortment rate.

**Results:**

The general patterns of reassortment are similar among five internal segments with the exception of segment 8, encoding the Non-Structural genes, which has a more divergent phylogeny. However, significant variation in rates between subtypes was observed. In particular, hemagglutinin-encoding segments of subtypes H5 to H9 reassort at a lower rate compared to those of H1 to H4, and Neuraminidase-encoding segments of subtypes N1 and N2 reassort less frequently than N3 to N9. Both host species and d_N_/d_S_ ratio were significantly associated with reassortment rate, while evolutionary rate was not associated. The d_N_/d_S_ ratio was negatively correlated with reassortment rate, as was the number of negatively selected sites for all segments.

**Conclusions:**

These results indicate that overall selective constraint and host species are both associated with reassortment rate. These results together identify the wild bird population as the major source of new reassortants, rather than domestic poultry. The lower reassortment rates observed for H5N1 and H9N2 may be explained by the large proportion of strains derived from domestic poultry populations. In contrast, the higher rates observed in the H1N1, H3N8 and H4N6 subtypes could be due to their primary origin as infections of wild birds with multiple low pathogenicity strains in the large avian reservoir.

## Background

Influenza A viruses are highly adaptable, able to evade host immune responses and to transmit among different host species. The segmented genome is composed of eight negative sense RNA strands. Segments 4 and 6 encode haemagglutinin (HA) and neuraminidase (NA), respectively. The external domains of HA and NA are the major targets for host neutralizing antibodies and are used in the subtyping of influenza viruses. The other six are internal gene segments: the first three segments encode the viral polymerase complex: PB2, PB1 and PA, and segment 5 encodes the nucleoprotein (NP). These four proteins comprise the ribonucleoproteins (vRNPs), which are the core of the virion, with the viral negative stranded RNAs packaged inside. Segment 7 encodes both matrix protein (M1) and ion channel protein (M2). Two separate Non-Structural proteins NS1 and NS2 are encoded by segment 8 [1,2].

The structure of the virus genome allows for exchange among the eight RNA segments between viruses co-infecting a cell, a process termed reassortment [3]. The viral reassortants derived from different virus subtypes can acquire completely new antigens (antigenic shift) thus avoiding recognition by previously infected hosts and allowing for efficient transmission, leading to a pandemic [4]. Three human influenza pandemics in the 20th century had either complete or partial avian origins: the H1N1 Spanish flu in 1918 appears to have been derived from an earlier avian source and both the H2N2 Asian flu in 1957 and the H3N2 Hong Kong Flu in 1968 were reassortants between human and avian strains [5-7]. Genetic reassortment also played a prominent role in the origin of the swine-origin influenza A (H1N1) 2009 virus [8].

Wild avian species of wetlands and aquatic environments such as the Anseriformes (particularly ducks, geese, and swans) and Charadriiformes (particularly gulls, terns, and waders) have been recognized as the major natural reservoirs of influenza A viruses where the viruses are maintained predominantly by asymptomatic birds, indicating a long-standing history of endemic infection [1]. Terrestrial poultry, such as chickens and turkeys are not traditionally seen as reservoir hosts of avian influenza virus (AIV), but are susceptible to infection with wild-bird-derived AIV [9] through exposure to fecal material from wild birds when they migrate through an area [10]. Previous studies indicated that domestic ducks may act as intermediaries between migratory ducks and terrestrial poultry in southern China [11,12], thus they could be thought as part of the larger reservoir within which different subtypes of influenza interact with each other [13].

Avian influenza A virus strains are classified as low pathogenicity (LP) or high pathogenicity (HP) [5,14]. Highly pathogenic strains primarily arise in subtypes H5 and H7 and are distinguished by the appearance of a highly basic cleavage site in HA [15,16]. Genetic exchanges between viruses circulating in wild and domestic birds have been documented, usually involving LPAI viruses in domestic poultry [17,18]. More rarely, HPAI virus can be found to infect both wild and domestic birds, as seen in the case of HPH5N1 avian influenza. Since 2003, HPAI H5N1 AIV spread from south China into Southeast Asia, causing sporadic transmissions to humans and tremendous damage to the poultry industry [19,20]. Repeated spill-over of Asian HPAI H5N1 viruses from domestic to wild birds has also been observed, which demonstrates the potential for reverse flow [21,22], and supports a close interaction between influenza strains in wild and domestic birds [23].

The 16 HA subtypes and 9 NA subtypes have allowed at least 103 of the possible 144 type A influenza A virus HA-NA combinations to arise [24], which indicates a high frequency of reassortment among different HA and NA subtypes. However some subtypes are rarely detected, indicating that some restrictions on possible combinations may exist. Thus, two important questions, regarding the general pattern of genetic interaction between different influenza A virus subtypes remain: whether certain subtypes are more likely to reassort than the other subtypes, and why this might be so.

Recent advances in Bayesian phylogenetics have provided methods which directly link patterns of genetic diversity to ecological processes, including changes in population size and substructure through time, allowing simultaneous insights into the spatial, temporal, and demographic dynamics of rapidly evolving pathogen populations, collectively described as “phylodynamics” [25-27]. The spatial phylodynamic process can be recovered from genomic data using phylogeographic analyses, allowing us to quantitatively study how and to what extent ecological features shape pathogen genetic diversity [28].

Unlike the dynamics of seasonal human influenza strains, characterized by a few co-circulating subtypes, little inter-subtype reassortment and broadly correlated evolution of lineages of the individual segments [29], avian influenza has many co-circulating subtypes which caused a complex distribution of internal segment lineages [30,31]. Conversely internal segment lineages have their own continuous histories, but with many different external segment partners [32]. Here we will consider the patterns of acquisition and loss of the external protein coding segments on the backbone of each internal protein coding segment, since this represents the mechanism for generating antigenic novelty in a circulating population of viruses from the perspective of the internal protein coding segments.

With the aim of exploring the rate of genetic exchange among viral subtypes during evolution, we carried out a phylodynamic analysis of the influenza A viruses circulating in avian populations (in both wild birds and domestic birds) from 1956 – 2011. In particular, we focused on investigating how avian influenza segments specific to the HA, NA and combined HA-NA subtypes evolve on the backbones of the six internal gene segments, by quantifying the inter-subtype reassortment rates and testing possible ecological and evolutionary factors that are associated with such pattern of reassortment: the relative proportion of each host species, the inferred evolutionary relaxed clock rates and the selective constraint acting on the genes.

## Results

### Time-scaled phylogenies of internal genes of Eurasian AIV

To explore how the HA and NA subtypes reassorted with the internal segments of Eurasian AIV, we used discrete trait models (in BEAST) upon empirical phylogenetic trees of the six internal gene segments. For reasons of computational tractability, we analyzed subsamples of 344 sequences generated using a stratified approach which maintained the range of genetic and subtype diversity. These were composed of 7 HA and 7 NA subtypes and 13 combined HA-NA subtypes respectively (see Methods and Additional file 1: Figure S2).

**Figure 1:**
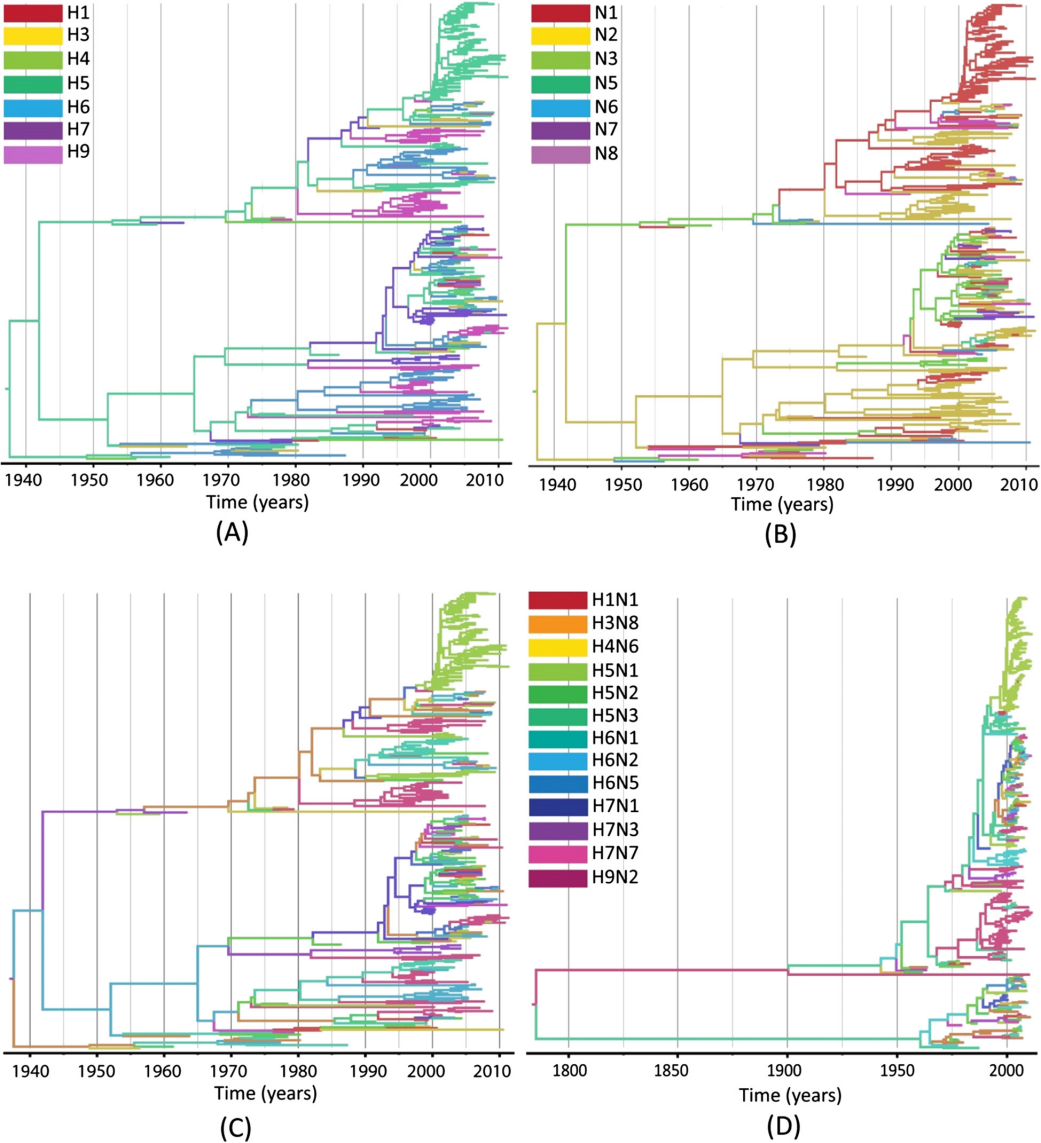
**Bayesian maximum clade credibility phylogenies for the internal segments encoding PB2 and NS. A**: PB2 phylogeny, colored by HA subtype. **B**: PB2 phylogeny colored by NA subtype. **C**: PB2 phylogeny colored by combined HA-NA subtype. **D**: NS phylogeny colored by combined HA-NA subtype. Branches are colored according to the different subtypes of their descendent nodes (C and D share the same key). The full tree with all taxon names is given in Additional file 1: Figure S2.

The reassortment history of Eurasian AIV was inferred by discrete trait models (HA, NA and combined HA-NA subtypes) on phylogenetic trees. Figure 1 shows the MCC tree of PB2 with branches colored by the HA subtype at the child nodes (or tips), and the other trees can be found in Additional file 1: Figure S2. The two most over-represented subtypes can be easily seen to form single subtype clades; colored green (H5N1) and orange (H9N2). However, it is apparent that some different subtypes were interspersed with each other, and the internal segments did not form monophyletic lineages with respect to antigenic subtype, indicating extensive reassortment between external and internal gene segments. A separation of two main clades can be seen in the PB2 phylogeny (Figure 1A-C). The sequences in each clade cover a range of subtypes, host, sample location and time and do not correspond to the strains belonging to allele A and B in NS (fewer than 40% of the sequences in either lineage are the same). In contrast, lower genetic diversity was observed in the remaining 4 gene segments (PB1, PA, NP and M) (Additional file 1: Figure S2).

### Reassortment rates of discrete subtypes of Eurasian AIV

A range of inter-subtype reassortment rates was found with respect to the internal segments of Eurasian AIV. For both HA and NA subtypes, there were 7 distinct states and thus each discrete trait model contained 42 (asymmetric) transition pairs (e.g. H1 to H3, H3 to H1, N1 to N2, N2 to N1). For the combined HA-NA subtypes, there were 156 different possible transition pairs based on 13 distinct states (e.g. H1N1 to H3N8, H3N8 to H1N1). We analyzed the inter-subtype reassortment rate at three levels: rate per segment (Table 1), mean rate per subtype (Table 2) and absolute transition rate between pairs of subtypes (Additional file 2: Table S2).

**Table 1:** Overall reassortment rate of six internal segments for H, N and HN combined subtype

	**PB2**	**PB1**	**PA**	**NP**	**M**	**NS**
H	0.30 (0.13,0.39)	0.30 (0.13,0.45)	0.31 (0.11,0.44)	0.28 (0.13,0.38)	0.37 (0.17,0.46)	0.63 (0.28,0.70)
N	0.46 (0.22,0.57)	0.47 (0.18,0.63)	0.49 (0.19,0.64)	0.29 (0.14,0.35)	0.40 (0.29,0.49)	0.72 (0.43,0.97)
H-N	0.37 (0.21,0.45)	0.46 (0.26,0.52)	0.37 (0.21,0.45)	0.34 (0.21,0.40)	0.35 (0.20,0.43)	0.68 (0.41,0.76)

**Table 2:** Inter-subtype reassortment rate per subtype for H, N and H-N combined subtypes of 6 internal segments

	**PB2**	**PB1**	**PA**	**NP**	**M**	**NS**
**H**						
H1	0.47	0.54	0.39	0.45	0.54	1.15
H3	0.35	0.35	0.35	0.29	0.41	0.65
H4	0.44	0.46	0.53	0.41	0.53	0.78
H5	0.06	0.04	0.05	0.02	0.03	0.06
H6	0.09	0.10	0.11	0.09	0.12	0.17
H7	0.16	0.23	0.14	0.18	0.14	0.26
H9	0.02	0.05	0.04	0.04	0.03	0.04
**N**						
N1	0.06	0.07	0.07	0.04	0.06	0.05
N2	0.09	0.07	0.10	0.07	0.12	0.16
N3	0.29	0.28	0.29	0.20	0.35	0.59
N5	0.58	0.48	0.64	0.36	0.75	1.01
N6	0.72	0.58	0.69	0.37	0.76	1.06
N7	0.57	0.84	0.59	0.39	0.56	0.99
N8	0.51	0.42	0.49	0.28	0.60	0.86
**H-N**						
H1N1	0.48	0.60	0.41	0.45	0.46	0.90
H3N8	0.34	0.39	0.34	0.29	0.33	0.64
H4N6	0.45	0.54	0.47	0.38	0.43	0.77
H5N1	0.03	0.05	0.04	0.03	0.03	0.06
H5N2	0.35	0.46	0.38	0.31	0.32	0.66
H5N3	0.35	0.54	0.45	0.36	0.42	0.75
H6N1	0.10	0.16	0.15	0.09	0.12	0.16
H6N2	0.21	0.29	0.18	0.18	0.18	0.51
H6N5	0.46	0.44	0.40	0.29	0.42	0.84
H7N1	0.36	0.41	0.29	0.34	0.29	0.59
H7N3	0.22	0.16	0.17	0.21	0.16	0.25
H7N7	0.40	0.60	0.41	0.41	0.33	0.81
H9N2	0.02	0.05	0.04	0.03	0.03	0.05

In general, the overall reassortment rate at the segment level was slightly higher for the NA subtypes than for the HA and HA-NA combined subtypes (Table 1). In addition, the reassortment rates of the combined HA-NA subtypes (e.g. H5N1) were related to the rate of the corresponding independent HA (e.g. H5) and NA (e.g.N1) subtypes with P < 0.01 in all six segments.

When comparing the reassortment rates among the six internal segments, we found the rates for five (PB2, PB1, PA, NP and M) were similar. Among these NP had a slightly lower and PB1 slightly higher overall rate for HA, NA and the HA-NA combined subtypes. In contrast to these five segments, the NS segment possessed significantly higher overall reassortment rates than those of the other segments (Table 1).

We compared the mean reassortment rates of different subtypes, and found a substantial range among each subtype in all six internal segments and all three subtype traits (Table 2). Specifically, of the HA subtypes, H5, H6, H7 and H9 showed lower reassortment rates relative to the others, and H5 and H9 had the lowest rates in the six internal segments. By contrast, the reassortment rates of the H1, H4, and H3 subtypes were high, especially for H1 with the NS segment, which had the highest rate (1.148 exchanges per year) compared to all the others. For NA subtypes, N1 and N2 presented lower reassortment rates compared to those of N3, N5, N6, N7 and N9. For the HA-NA joint subtypes, reassortment rates of H5N1 and H9N2 were considerably lower than with the other subtypes in all segments, and H9N2 had the lowest rates in the PB2 segment (0.0220 exchanges per year) and NS segment (0.0487 exchanges per year), while H5N1 had the lowest rates in the other segments (PB1: 0.0449; PA: 0.0352; NP: 0.0249; and M: 0.0266 exchanges per year); other subtypes with low rates were H6N1, H6N2 and H7N3 subtypes. In contrast, H1N1, H3N8 and H4N6 and others exhibited high reassortment rates.

**Figure 2:**
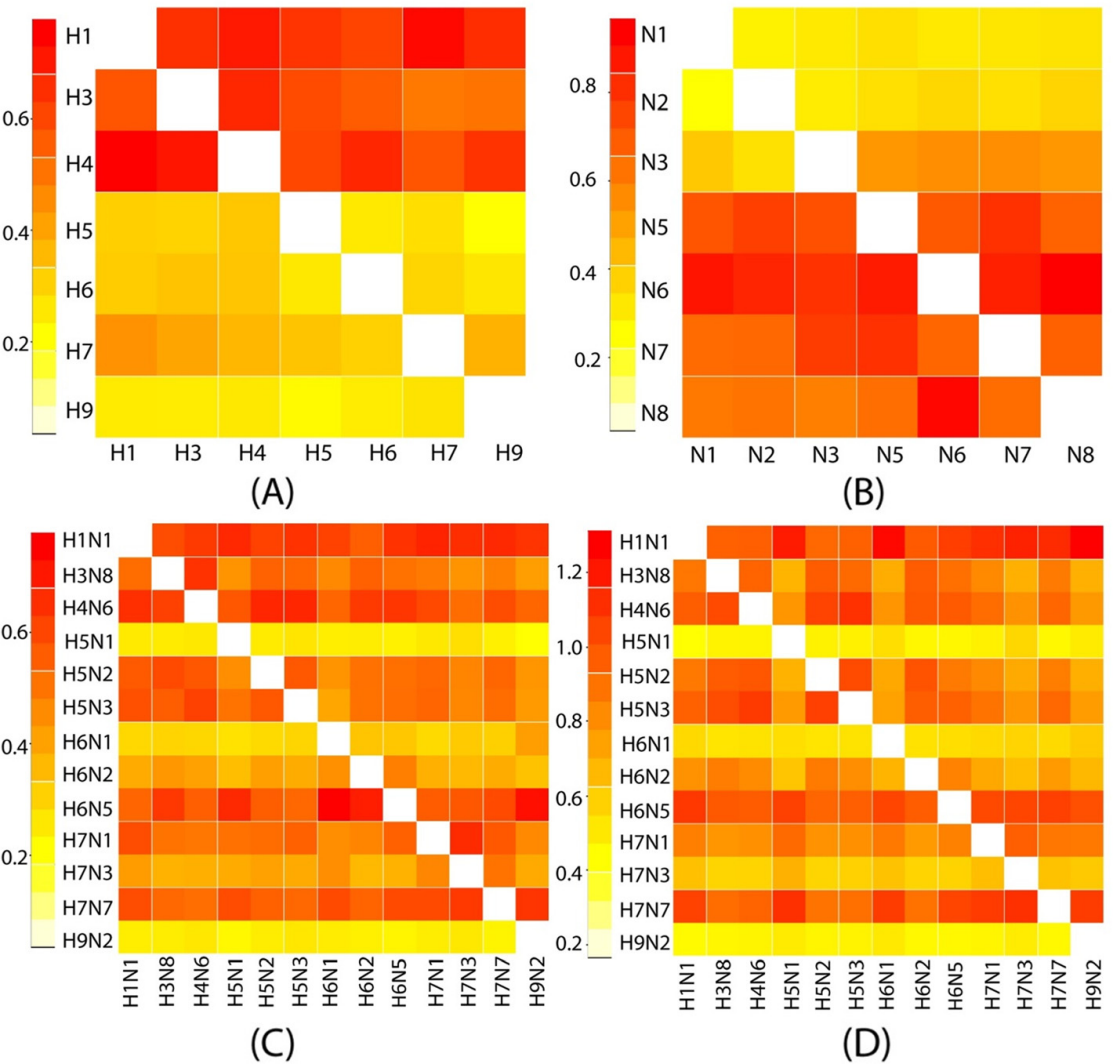
**Reassortment rates between internal segments of different subtypes. A**: PB2 segments associated with HA subtypes shown, which means PB2 that are associated with H1, H3 and H4 subtypes rapidly reassort to become associated with any HA, while PB2 associated with H5, H6, H7 and H9 subtypes reassort more slowly to become associated with any HA. **B**: PB2 segments associated with NA subtypes shown; **C**: PB2 segments associated with combined HA- NA subtypes shown; **D**: NS segments associated with combined HA- NA subtypes shown. Reassortment rates are shown as a heat map. Both forward and reverse rates are shown, with subtypes labeled on the left side acting as donors and subtypes labeled at the bottom acting as recipients. The values of the mean reassortment rates, in exchanges per year, are represented as colors from yellow (low) to red (high). The scale of rates is on the left hand side of each plot.

For clarity, the patterns of pairwise transition between individual subtypes are represented as a series of heat maps for the six internal segments in Figure 2 (and Additional file 1: Figure S5). The inferred asymmetric reassortment transition rate matrixes are shown in Additional file 2: Table S2. The color intensity in each column reflects the rate with which each subtype (labeled on the y-axis as donors) was found to reassort into a clade of other subtypes. The color intensity in each row reflects the rate with which the reciprocal events were inferred (labeled on x-axis as recipients).

A range of reassortment rates were observed among subtypes as recipients, which indicates that each subtype possesses a different probability of giving the viral gene to viruses of other subtypes during co-infection. In comparison, the rates of subtypes as donors from the other subtypes were consistent which gives rise to the horizontal striping seen in the heat maps. The distinction between source and sink rates were further quantified by estimating the variance of rates in columns to the variance of rates in rows (Additional file 2: Table S2, Additional file 1: Figure S6), which showed the variances were significantly smaller in the source to sink direction than the reverse. This pattern can be seen in the HA, NA subtypes independently and in the HA-NA combined subtypes, and is also similar in all six internal segments.

### Correlation of reassortment with genetic diversity

To further explore what factors are required to explain the pattern of reassortment rate in the segment level, we first tested the genetic diversity at the segment level which can be represented by the mean time to the most recent common ancestor (tMRCA) based on each phylogeny. The tMRCA of three internal gene segments were quite similar and recent: PB2 (75.71 years, with 95% HPD (Highest Posterior Density): 64.70-87.21), PA (77.95 years with 95% HPD: 64.42-93.01) and the M segment (73.17 years, with 95% HPD of 61.56-87.87). The PB1 segment had an older mean tMRCA, of 105.12 years (95% HPD: 80.21-135.71) while NP was less divergent than the other segments (tMRCA 63.22 years; 95% HPD: 58.32-69.33). Compared to the other segments, the tree topology of the NS segment was characterized by a very deep divergence between the two sub-lineages formed by the A and B alleles which represent two distinct gene pools, and consequently has a much older tMRCA of 226 years (95% HPD: 112–273) (Figure 1D).

**Figure 3:**
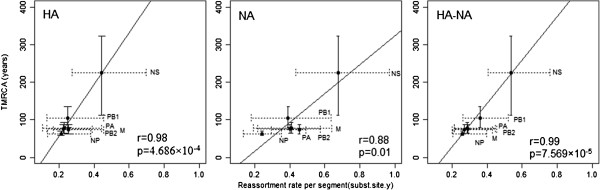
**Correlation between TMRCA (years) and reassortment rate (exchanges per year).** For HA subtype, NA subtype and HA-NA combined subtype, respectively. Scatterplots of mean TMRCA (95% HPD intervals were shown in solid vertical lines) against the mean reassortment rate (95% HPD intervals shown in dotted horizontal lines) of each internal segment. The related segment names were labelled alongside.

The association between genetic diversity and the overall reassortment rate of six internal segments of Eurasian AIV was tested for HA, NA and HA-NA combined subtypes, respectively. The results (Figure 3) exhibited a strong positive correlation for the H subtype (p = 4.7 × 10^-4^, R = 0.982), the N subtype (p = 0.01, R = 0.879) and the H-N combined subtype (p = 7.569 × 10^-5^, R = 0.99).

### Correlation of reassortment rates with host

**Figure 4:**
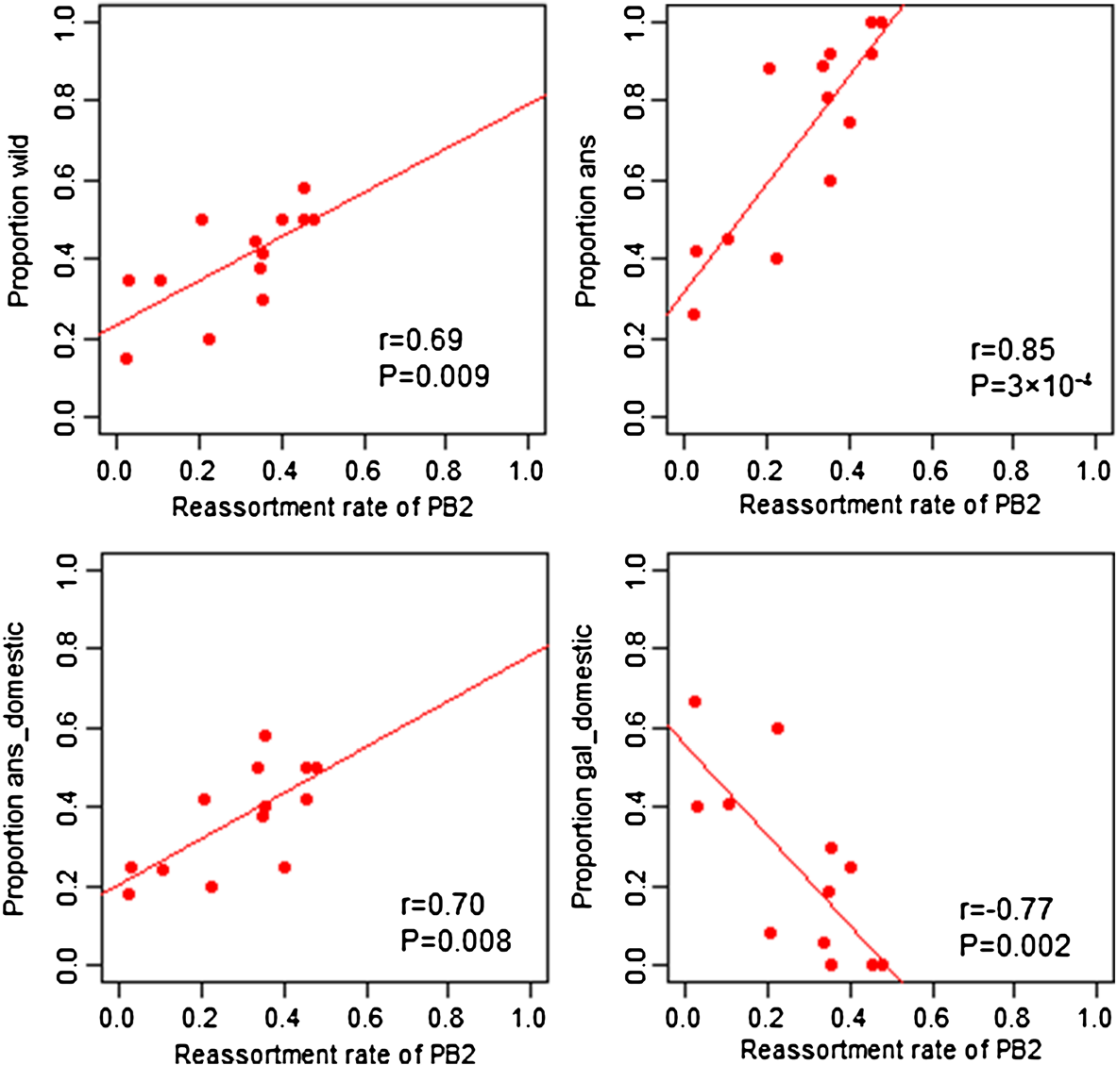
**Correlation between prevalence in host and reassortment rate per subtype in PB2 segment.** The scatterplot of the mean reassortment rates of 13H-N combined subtypes of each internal segment against the proportion of AIV 1) in wild birds, 2) in Anseriformes 3) in domestic birds of Anseriformes and 4) in domestic birds of Galliformes. The plots of the remaining five segments are shown in supplementary figures (Additional file 1: Figure S8-S11).

The prevalence of AIV in different avian hosts could also influence the reassortment pattern. The host prevalence of different subtypes was calculated from the subsampled data sets used to make the phylogenetic trees, which was proportional to the original data. The primary host difference lies between wild birds and domestic birds which live in completely different ecosystems. Subtypes H1N1, H3N8 and H4N6 had the highest prevalence in wild birds (Table 3). We tested the association between individual reassortment rates and the proportion of domestic birds for the HA-NA combined subtypes. We found that there was a significant positive correlation between the proportion of wild bird strains per subtype and the reassortment rates for those subtypes, and that the correlation held in each of the six segments (PB2: p = 0.009, R = 0.69; PB1: p = 0.004, R = 0.73; PA: P = 0.006, R = 0.71; NP: P = 0.02, R = 0.62; M: P = 0.06, R = 0.71; NS: p = 0.002, R = 0.76; Figure 4A and Additional file 1: Figure S8).

**Table 3:** Proportion of domestic birds of each subtype analyzed

**Subtype**	**Wild**^**a**^	**Ans**^**b**^	**Ans-domestic**^**c**^	**Gal-domestic**^**d**^
H1N1	0.50	1.00	0.50	0.00
H3N8	0.44	0.89	0.50	0.06
H4N6	0.58	0.92	0.42	0.00
H5N1	0.35	0.42	0.25	0.40
H5N2	0.38	0.81	0.38	0.19
H5N3	0.42	0.92	0.58	0.00
H6N1	0.34	0.45	0.24	0.41
H6N2	0.50	0.88	0.42	0.08
H6N5	0.50	1.00	0.50	0.00
H7N1	0.30	0.60	0.40	0.30
H7N3	0.20	0.40	0.20	0.60
H7N7	0.50	0.75	0.25	0.25
H9N2	0.15	0.26	0.18	0.67

^a^The overall proportion of AIV with a wild bird host.

^b^The overall proportion of AIV with a host species belonging to Anseriformes.

^c^The proportion of AIV with a domestic Anseriformes host.

^d^The proportion of AIV with a domestic Galliformes host.

The prevalence of AIV in different host species (by Order) was also analyzed. In this dataset, subtypes with a high reassortment rate also have the highest prevalence in Anseriformes, which is the Order representing birds living in predominantly freshwater habitats (Additional file 2: Table S4). We found a very strong positive correlation between reassortment rates and the proportion of Anseriformes (PB2: p = 3 × 10^-4^, R = 0.85; PB1: p = 2 × 10^-4^, R = 0.84; PA: p = 2 × 10^-4^, R = 0.84; NP: p = 0.002, R = 0.77; M: p = 4 × 10^-5^, R = 0.89; NS: p = 9 × 10^-6^, R = 0.90) (Figure 4B and Additional file 1: Figure S9), which may indicate the subtype with high reassortment rate could be better explained by their high proportion in waterfowl than wild birds. We further tested wild (mainly mallard in our study) and domestic Anseriformes (mainly domestic duck in our study) separately (Table 3). Both of them show a strong positive correlation with reassortment rate per subtype (Additional file 1: Figure S10 and Additional file 1: Figure S11).

On the other hand, subtypes H9N2, H7N3, H7N1 and H5N1 had a high prevalence in domestic birds of the order Galliformes (chickens, turkeys, etc.). There was a strong negative correlation between proportion of AIV in domestic birds of the order Galliformes and rate of reassortment per subtype (PB2: p = 0.002, R = −0.77; PB1: p = 0.001, R = −0.79; PA: p = 0.001, R = −0.70; NP: p = 0.008, R = −0.70; M: p = 2 × 10^-4^, R = −0.84; NS: p = 1 × 10^-4^, R = − 0.85) (Figure 4C and Additional file 1: Figure S12).

### Correlation of reassortment rate with evolutionary rate

We further tested whether the reassortment rates were correlated with evolutionary rates of AIV at both segment and subtype level. The overall evolutionary rates of Eurasian AIV in different segments were the relaxed clock rates per segment from the MCMC tree (Table 4) [25]. No significant difference in evolutionary relaxed clock rate was found among six internal genes of the analyzed AIV sequences, with estimated mean rates from 2.27 to 2.85 × 10^-3^ substitutions per site per year (subs/site/year). In addition, no significant correlation was identified between overall reassortment per segment and evolutionary rate per segment (P = 0.7).

**Table 4:** Evolutionary Rate of internal segment of Eurasian AIV

	**PB2**	**PB1**	**PA**	**NP**	**M**	**NS**
segment^a^	2.85 (2.54,3.19)	2.27 (1.96,2.61)	2.62 (2.32,2.91)	2.65 (2.36,2.95)	2.41 (2.07,2.76)	2.56 (2.18,2.95)
subtype^b^						
H1N1	2.63 (1.88,3.38)	2.36 (1.62,3.10)	2.52 (1.41,3.64)	2.70 (1.69,3.72)	2.62 (1.32,3.93)	2.28 (1.46,3.11)
H3N8	2.40 (1.87,2.93)	2.86 (2.12,3.60)	2.38 (1.62,3.13)	2.30 (1.61,2.99)	2.13 (1.33,2.92)	2.20 (1.60,2.81)
H4N6	2.80 (2.42,3.18)	2.59 (1.85,3.33)	2.33 (1.50,3.15)	2.62 (1.73,3.51)	2.28 (1.39,3.17)	2.56 (1.80,3.31)
H5N1	2.93 (2.61,3.25)	2.84 (2.10,3.58)	2.62 (1.85,3.38)	2.60 (1.94,3.26)	2.32 (1.60,3.04)	2.73 (2.30,3.16)
H5N2	2.91 (2.28,3.54)	2.78 (2.04,3.52)	2.39 (1.57,3.21)	2.74 (1.86,3.62)	2.50 (1.57,3.43)	2.45 (1.83,3.07)
H5N3	3.01 (2.29,3.72)	2.44 (1.70,3.18)	2.42 (1.54,3.30)	2.54 (1.66,3.43)	2.68 (1.51,3.85)	2.25 (1.61,2.88)
H6N1	3.20 (2.67,3.74)	2.78 (2.04,3.52)	2.54 (1.71,3.36)	2.53 (1.81,3.24)	2.16 (1.40,2.93)	2.61 (2.03,3.19)
H6N2	2.85 (2.32,3.38)	2.90 (2.16,3.64)	2.61 (1.78,3.45)	2.52 (1.79,3.25)	2.51 (1.59,3.43)	2.51 (1.88,3.14)
H6N5	2.72 (1.97,3.47)	2.99 (2.25,3.73)	2.45 (1.55,3.34)	2.54 (1.68,3.41)	2.18 (1.16,3.20)	2.30 (1.47,3.13)
H7N1	2.77 (2.08,3.45)	2.68 (1.94,3.42)	2.35 (1.47,3.24)	2.57 (1.71,3.44)	2.37 (1.32,3.41)	2.33 (1.57,3.09)
H7N3	2.54 (1.89,3.18)	2.42 (1.68,3.16)	2.08 (1.31,2.85)	2.47 (1.66,3.28)	2.17 (1.19,3.16)	1.92 (1.25,2.59)
H7N7	2.98 (2.15,3.80)	2.55 (1.81,3.29)	2.31 (1.39,3.22)	2.46 (1.59,3.32)	2.06 (1.07,3.05)	2.32 (1.35,3.28)
H9N2	3.11 (2.71,3.52)	2.87 (2.13,3.61)	2.74 (1.92,3.56)	2.72 (2.00,3.45)	2.36 (1.61,3.11)	2.62 (2.18,3.05)

^a^Estimated overall evolutionary rate (×10^3^ subst/site/year) per internal segment. Mean rate under the uncorrelated log-normal relaxed molecular clock with 95% HPD of all MCMC samples are shown in brackets.

^b^Estimated evolutionary rate (×10^3^ subst/site/year) per H-N combined subtype in each internal segment. Mean clock rate of certain subtype on MCMC tree nodes with 95% confidential interval (CI) of all MCMC samples are shown in brackets.

The evolutionary rates in different subtypes were estimated by considering the relaxed clock rates corresponding to branches of particular subtypes. The rates per subtype were also similar between different subtypes in the same segment and not significantly different across different segments (Table 4). There was no significant correlation between evolutionary rate and reassortment rate per subtype in each internal segment (P > 0.1) (Additional file 1: Figure S13), which is also confirmed by the distribution of correlation coefficients between the evolutionary rates (relaxed clock rates from 1000 random MCMC samples post-burnin) and reassortment rates (rates were taken from 1000 random MCMC samples post-burnin). In addition, there was no indication of any interaction between evolutionary rate and the proportion of domestic birds (P > 0.5).

### Correlation of reassortment with selection

**Figure 5:**
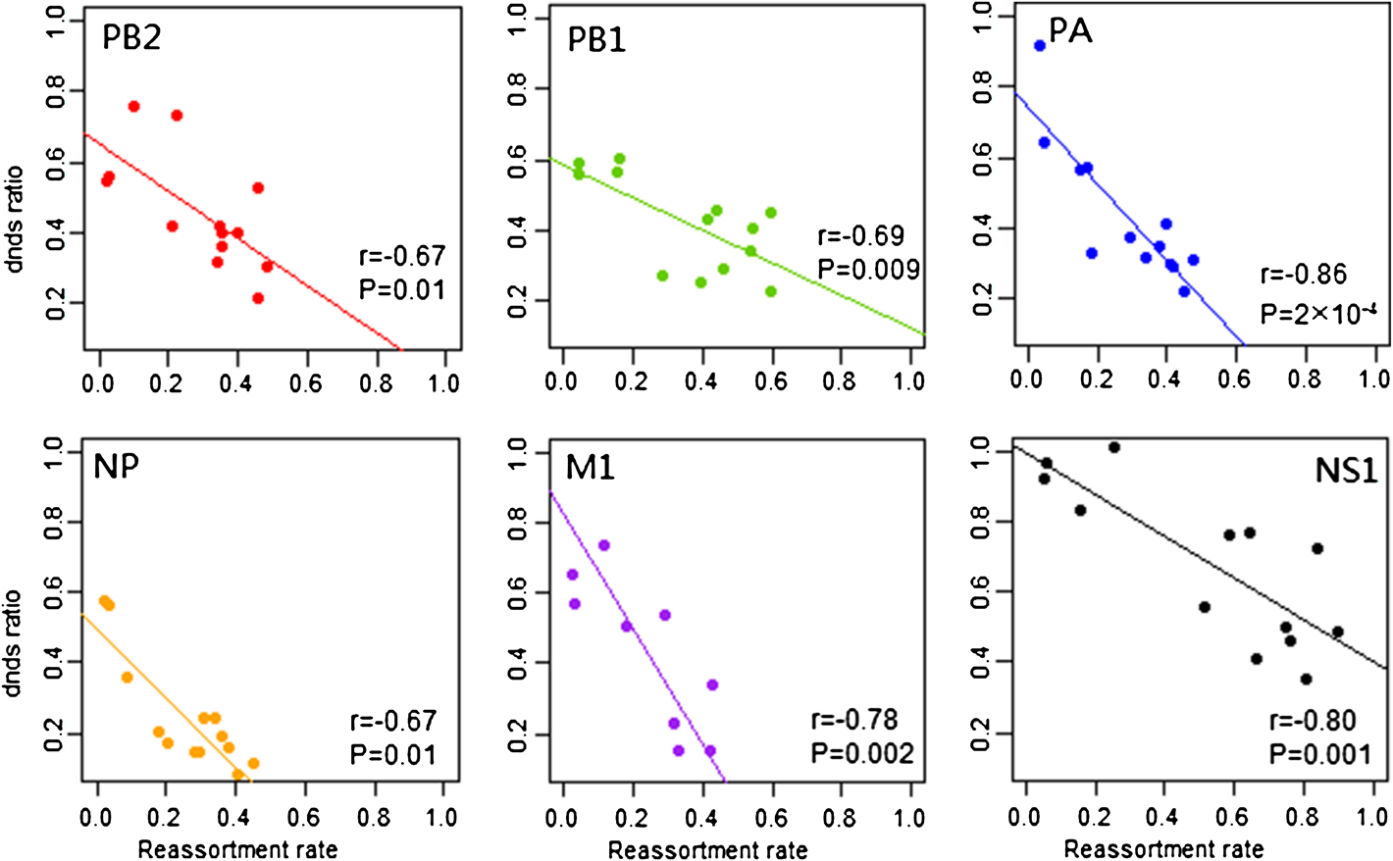
**Correlation between selection and reassortment rate.** The scatterplot of reassortment rate of HA-NA combined subtype against dN/dS ratio of 6 internal segments were represented by different colors: PB2 (red); PB1 (green); PA (blue); NP (orange); M1 (purple); NS1 (black).

Finally, we estimated the total d_N_/d_S_ ratio for each internal segment as well as the individual ratio per subtype in each segment (Table 5). Initial analysis of the coding regions in the five segments PB2, PB1, PA, NP and M1 showed the d_N_/d_S_ ratios were similar to each other with mean values from 0.11 to 0.17, indicating, as expected, that at the amino acid level purifying selection predominates in these internal segments; however, the ratio for the NS segment (mean 0.439) was significantly higher than those of the other segments. This suggested a strong correlation between reassortment rate and d_N_/d_S_ ratio at the segment level (P = 0.006, R = 0.93). However, this result is entirely dependent on the high value for NS1 and the correlation became negative and non-significant when NS1 was left out (R = −0.23, p = 0.83). Compared to the evolutionary rate, the d_N_/d_S_ ratio for each subtype differs noticeably between different subtypes in the same segment and is very different across different segments (Table 4). For instance, in PB2, H4N6 had the lowest d_N_/d_S_ ratio (0.22, 95% HPD: 0.13-0.30) and H6N1 had the highest (0.76, 95% HPD: 0.55-0.96). In NS1, H7N7 had the lowest ratio (0.351, with 95% HPD: 0–0.842) and H7N3 had the highest ratio which was equal to 1, but this difference was not significant given a wide range of statistical uncertainty for these subtypes in the NS1 segment. We found strong negative correlations between mean d_N_/d_S_ and the reassortment rate per subtype in each of the six segments (PB2: p = 0.01, R = −0.67; PB1: p = 0.009, R = −0.69; PA: P = 2 × 10^-4^, R = −0.86; NP: P = 0.01, R = −0.67; M: P = 0.002, R = −0.78; NS: p = 0.001, R = −0.80) (Figure 5).

**Table 5:** **d**_**N**_**/d**_**S **_**ratio of internal segments of Eurasian AIV**

	**PB2**	**PB1**	**PA**	**NP**	**M1**	**NS1**
segment^a^	0.14 (0.14,0.15)	0.12 (0.12,0.13)	0.17 (0.17,0.17)	0.13 (0.13,0.14)	0.11 (0.11,0.12)	0.59 (0.15,1.03)
subtype^b^						
H1N1	0.31 (0.15,0.46)	0.23 (0.09,0.37)	0.30 (0.16,0.43)	0.64 (0.11,0.38)	0.02 (0.02,0.11)	0.49 (0.06,0.91)
H3N8	0.31 (0.19,0.43)	0.25 (0.16,0.34)	0.31 (0.21,0.42)	0.35 (0.14,0.24)	0.15 (0.03,0.28)	0.77 (0.39,1.15)
H4N6	0.22 (0.13,0.30)	0.41 (0.26,0.55)	0.31 (0.19,0.43)	0.47 (0.16,0.31)	0.34 (0.06,0.62)	0.46 (0.15,0.78)
H5N1	0.56 (0.48,0.64)	0.56 (0.48,0.65)	0.92 (0.80,1.04)	0.80 (0.57,0.69)	0.65 (0.48,0.82)	0.97 (0.78,1.15)
H5N2	0.42 (0.29,0.54)	0.29 (0.20,0.37)	0.35 (0.24,0.46)	0.49 (0.24,0.36)	0.23 (0.09,0.37)	0.41 (0.16,0.65)
H5N3	0.36 (0.22,0.49)	0.34 (0.20,0.48)	0.22 (0.13,0.31)	0.51 (0.19,0.35)	0.15 (0.02,0.29)	0.50 (0.15,0.84)
H6N1	0.76 (0.55,0.96)	0.60 (0.45,0.75)	0.57 (0.39,0.74)	0.64 (0.36,0.50)	0.73 (0.38,1.08)	0.83 (0.56,1.11)
H6N2	0.42 (0.29,0.54)	0.27 (0.20,0.35)	0.33 (0.24,0.41)	0.43 (0.21,0.32)	0.50 (0.25,0.75)	0.56 (0.32,0.80)
H6N5	0.53 (0.14,0.92)	0.46 (0.22,0.69)	0.41 (0.20,0.62)	0.77 (0.15,0.46)	0.04 (0.04,0.25)	0.72 (0.12,1.32)
H7N1	0.40 (0.21,0.59)	0.43 (0.19,0.67)	0.37 (0.18,0.56)	0.98 (0.24,0.61)	0.53 (0.08,1.14)	0.76 (0.12,1.40)
H7N3	0.73 (0.08,1.39)	0.57 (0.33,0.80)	0.57 (0.28,0.86)	0.95 (0.17,0.56)	1.05 (0.21,1.89)	1.01 (0.07,2.43)
H7N7	0.40 (0.19,0.61)	0.45 (0.20,0.71)	0.29 (0.16,0.42)	0.52 (0.08,0.30)	0.03 (0.05,0.21)	0.35 (0.03,0.84)
H9N2	0.55 (0.46,0.64)	0.59 (0.52,0.67)	0.64 (0.56,0.72)	0.80 (0.56,0.68)	0.57 (0.40,0.74)	0.92 (0.72,1.12)

^a^Estimated overall d_N_/d_S_ ratio per internal segment. Mean ratio of each internal segments with 95% (CI) of all MCMC samples shown in brackets.

^b^Estimated d_N_/d_S_ ratio per H-N combined subtype in each internal segment. Mean d_N_/d_S_ of each subtype on MCMC tree nodes with 95% CI of all MCMC samples are shown in brackets.

In order to confirm the negative correlation between the d_N_/d_S_ ratio and inter-subtype reassortment rate seen in BEAST, we used the independent approach of Single Likelihood Ancestor Counting (SLAC) in the HyPhy package (http://www.datamonkey.org/) to estimate d_N_/d_S_ per subtype. We found a strong negative correlation between inter-subtype reassortment and d_N_/d_S_ ratio in all six internal segments using SLAC as well (Additional file 1: Figure S14). SLAC estimates the numbers of codons subject to positive and negative selection and we were thus able to identify the nature of the differences in selection. There was no evidence for positive selection contributing to the variation among subtypes, however there was substantial variation in the number of negatively selected sites, which was strongly negatively correlated with reassortment rate with R < −0.65, p ≤ 0.01, for all segments except NS1 for which R = −0.55, p = 0.05 (Additional file 2: Table S4). Therefore the intensity of purifying selection, or selective constraint, on the internal segments is strongly associated with the rate of reassortment.

In addition, we also found a strong negative correlation between the proportion of wild birds and the d_N_/d_S_ ratio of different subtypes per segment (PB2: p = 0.04, R = −0.58; PB1: p = 0.01, R = −0.46; PA: p = 0.04, R = −0.57; NP: p = 0.02, R = −0.64; M: p = 0.007, R = −0.71; NS: p = 0.002, R = −0.77). We then jointly estimated the partial correlation coefficients among d_N_/d_S_, host species and reassortment rates [33]. This showed that reassortment rate was still significantly negatively correlated with d_N_/d_S_ independent of the proportion of wild birds as host in all six internal segments (R: -0.79 to −0.44, P < 0.01; Additional file 2: Table S3 column 5 and 7); and independently positively correlated with proportion of wild birds as host (R:-0.66 to −0.29, P < 0.07; Additional file 2: Table S3 column 8 and 10) with borderline significance, thus both host and selective constraint are associated with reassortment rate.

## Discussion

We have systematically analyzed the evolution of internal segments of avian influenza viruses use a comprehensive avian influenza A virus dataset. Using Bayesian analysis of phylogenetic discrete traits within the integrated statistical framework in the BEAST software package, we quantified evolutionary parameters including reassortment rate, evolutionary relaxed clock rate and selective constraint for discrete. Notably, the quantified pattern of reassortment illustrates how and to what extent the subtypes of AIV interact in the internal segments in bird populations. Firstly, the overall inter-subtype reassortment rates are similar in five segments, while NS has higher rates overall, and secondly, in each segment, reassortment rates of different subtypes showed significant diversity and were asymmetric.

The reassortment rate of the six internal segments of Eurasian AIV is significantly associated with gene diversity, which is probably due to the phylogenetic history and the biological function of the segments during virus infection [34]. Here we identified that NS has a much more diverse phylogeny and higher inter-subtype reassortment rate than the other five segments of the avian influenza viruses of Eurasian lineage. This result is in agreement with those of a previous study on AIV from the wild bird population of North America, which found extensive phylogenetic incongruence in the NS gene [32]. The NS phylogeny of avian influenza viruses is characterized by a deep divergence between the A and B alleles suggesting that the two alleles are subject to some form of balancing selection [32,35-38].

While we found that reassortment rate was not associated with evolutionary rate, the segment-wide d_N_/d_S_ ratio per subtype was strongly negatively correlated with the inter-subtype reassortment. The variation in d_N_/d_S_ ratio was revealed by two independent methods: robust counting in BEAST [39], and SLAC in HyPhy [40]. The latter gives direct estimates of the numbers of selected sites from which it was seen that the variation between subtypes in d_N_/d_S_ appears to arise from differences in the extent of purifying selection, as indicated by differences in the number of negatively selected sites for each subtype, which itself yielded a strongly negative association with reassortment rate (SLAC identified only 3 positively selected sites; Additional file 2: Table S4). This suggests that the overall level of purifying selection, or selective constraint, differs between subtypes: more negatively selected sites indicating higher overall constraints leading to lower d_N_/d_S_.

As mentioned above, the evolutionary relaxed clock rates (of the internal gene segments) in Eurasian AIV show little difference among viral subtypes and also between the internal segments. In our results therefore, evolutionary rates were not correlated with inter-subtype reassortment rates and also not affected much by host species. This agrees with the conclusions of a previous study which indicated that the evolutionary rates in wild aquatic birds are not radically lower than those seen in poultry species [41,42]. Pathogenicity status might also be an explanation of the lower reassortment rates of H5N1: HPAI H5N1 is lethal to poultry [43] thus could have had less chance to undergo reassortment in the host due to the short duration of infection of the birds as compared to the less symptomatic infection, typically of longer duration, in low pathogenicity strains. However, our results indicate that pathogenicity status (which is defined in chickens) is not the major factor affecting the overall reassortment rate in all species; of those subtypes with high pathogenicity, such as H5N1, H7N3, H7N1 and H7N7, only H5N1 has low reassortment rate; and there are other AIV subtypes with low pathogenicity that have low reassortment rate, most notably H9N2 [44].

We show that the reassortment rates among viral subtypes are correlated with their prevalence in different types of host. High reassortment rates could be associated with high prevalence of certain subtypes in wild birds and particularly among Anseriformes host species. In contrast, the lower reassortment rate of certain subtypes could be explained by their high prevalence in domestic birds (mainly in Galliformes). Wild bird and domestic bird populations represent two different ecosystems: natural and artificial respectively, in which influenza viruses face different ecologic constraints such as host population structure density, and opportunities for virus transmission [45]. For instance, transmission by feces provides a way for wild birds to spread viruses to other wild birds as they migrate through an area [10], while AIV in domestic birds, especially domestic birds of the order Galliformes may exist in a more isolated population (within a physically confined flock) than might occur in wild birds, thus with less possibility of reassortment.

In comparison to the AIV in domestic Galliformes, AIV at high prevalence in the domestic duck (the H6 subtype in our study) has a higher reassortment rate. Domestic ducks or geese farming occurs in high-density but free-range settings in Asia [46]. This farming style creates an environment in which migratory birds and domestic ducks are in close contact, and provide more opportunities for AIV to reassort [47]. In addition, different responses to virus infection may also influence the opportunity of AIV reassortment in certain host species. Avian influenza virus in chickens (Galliformes) often leads to severe respiratory symptoms and high mortality rates of up to 100%. In contrast, AIV infection generally causes no major clinical signs and immune response in ducks (Anseriformes) [48]. Different influenza virus subtypes can also infect wild ducks concomitantly, creating the opportunity for genetic mixing [49]. Those subtypes showing the high reassortment rates in our study, especially H1N1, H3N8 and H4N6 represent the most common subtype combinations that are found in wild ducks during periods of peak prevalence and have a maximum detectable subtype diversity in the late summer and fall [32,50].

## Conclusions

In summary, we quantified the inter-subtype reassortment rates using discrete trait modeling upon time resolved phylogenies with respect to all six internal segments of Eurasian AIV. The overall reassortment rates for the external protein coding segments on internal protein coding segments were highly correlated with the internal segment genetic diversity (divergence time) and negatively correlated with d_N_/d_S_ ratio and selective constraint. In addition, we found that different subtypes are characterized by reassortment rates that are strongly dependent on host type (wild *v.* domestic). These results are consistent with the hypothesis that lack of immunological cross-protection at the subtype level in avian species allows frequent mixed infections in wild bird populations and provide novel insights into the evolutionary dynamics of avian influenza viruses.

## Methods

### Data preparation and Preliminary phylogenetic analysis

Initial analyses included 3226 full length complete genome avian influenza sequences of any subtype and sampling year from the NCBI Influenza Virus Resource (http://www.ncbi.nlm.nih.gov/genomes/FLU). Laboratory recombinants or highly cultured sequences were excluded.

The locations of samples were classified by countries and states/provinces separately. The host species were categorized by taxonomic bird Orders, and were further classified into wild birds and domestic birds: for instance, aquatic birds of the orders of Anseriformes (ducks, swans, etc.) and Charadriiformes (gulls, terns, etc.). Domestic birds can be further separated into domestic Anseriformes (ducks, geese etc.) and domestic Galliformes (chickens, turkeys etc.). The date of collection of each sequence was converted into fractional years (decimal date) for the estimation of divergence time, however for isolates where only year information was available the date was taken to be half way through the year, and if only month and year information was available the date was taken to be the 15^th^ of the month (shown in Additional file 2: Table S5)”. For each internal segment, sequence alignments of coding regions were created using MUSCLE [51] and then adjusted manually using BioEdit version 7.2.3 (http://www.mbio.ncsu.edu/bioedit/page2.html). The gene segment alignments were manually concatenated to generate a single alignment of complete genome sequences, and duplicate genomes (having 100% nucleotide identity) were removed.

A preliminary phylogenetic tree of each segment was generated using the Neighbor Joining method in MEGA 5. These phylogenies were analyzed using Path-O-Gen version 1.2 (http://tree.bio.ed.ac.uk/software/pathogen/), which is a tool to investigate the temporal signal and ‘clock-likeness’ of molecular phylogenies. The plots of root-to-tip showed a good linear relationship between genetic divergence and sampling time of the AIV sequences, except two strains (A/pintail/ALB/179/1993 in PB2 and A/shorebird/DE/12/2004 and A/duck/Yokohama/aq10/2003 in HA, respectively), indicating that they might contain incorrect date information and were removed from the dataset. Thus, our final dataset comprised 2996 full genome sequences sampled over 55 years from 1956 to 2011. Phylogenetic trees for each segment were generated using RAxML [52], employing maximum likelihood (ML) analyses with 500 bootstraps.

Phylogenetic analysis of the 2996 AIV sequences datasets confirms a clear separation of AIV sequences from east and west hemispheres, although some sequences are found to cross the boundaries, which confirmed that geographical separation of host species has shaped the avian influenza A virus gene pool into almost completely independently evolving Eurasian and American lineages (Additional file 1: Figure S1). We focused our study on the avian influenza sequences of the Eurasian lineage, which included 1140 strains and composed of 15 HA subtype (H1-16, without H14), 9 NA subtype and 60 combined HA-NA subtypes in total.

### Time-scaled phylogenetic analysis

Time-scaled trees were generated for each internal protein-coding segment with a subsample of the 1140 sequences dataset with known isolation dates using BEAST (version 1.7.3). Different substitution models, clock models and tree models were evaluated for each segment by the calculation of the Bayes factor (BF), which is the ratio of the marginal likelihoods of the two models, and log BF > 3 was taken as indicating support for one model over another [53] in Tracer v1.4 (http://tree.bio.ed.ac.uk/software/tracer/) [54]. A general-time-reversal (GTR) model [55] with gamma distributed rate heterogeneity of 4 rate categories (C4) was chosen for the M and NS segments for both of which have overlapping reading frames. For the PB2, PB1, PA and NP segments, the SRD06 nucleotide substitution model [56] was chosen as the default nucleotide substitution model (preferred over GTR model with log BF from 3242 to 3289), which allows one Hasegawa, Kishino and Yano (HKY) model [57] for codon positions 1 and 2 and a different HKY model for position 3, together with gamma-distributed rates across sites. A constant population size model (preferred to either an exponential model or a Bayesian skyride model with log BF from 7.23 to 7.52) and a relaxed uncorrelated log-normal molecular clock model (preferred to a strict clock model with log BF from 5.66 to 15.32) were chosen for all eight segments. The MCMC was run for 10^8^ steps and sampled every 10^4^ steps. Two independent runs were used in each segment to confirm the convergence and then combined. The Maximum Clade Credibility (MCC) trees were obtained and the mean ages of each node and the corresponding HPD were calculated by using Tree Annotator v1.7.3 in BEAST.

### Discrete trait mapping and estimation of reassortment rates

To quantify the genetic association among subtypes (HA, NA and HA-NA) in AIV from Eurasia, We implemented the asymmetric discrete traits model with irreversible transitions (with the uniform prior with mean = 1 for the trait.rate) to reconstruct the ancestral subtype states of the internal nodes from the posterior time-scaled tree distribution [29], using a relaxed uncorrelated log-normal molecular clock model, SRD60 model of nucleotide substitution and a constant-population coalescent process prior across the phylogeny of each segment. In addition, a BF test was constructed to identify the significant transition rates between discrete traits with the Bayesian stochastic search variable selection (BSSVS) extension of the discrete model. For the rates calculated from BSSVS, a BF test was adopted to identify significant non-zero transition rates (BF = 3) [28]. For each of the six internal segments, we did 3 separate analyses on sequences labelled by HA subtype, NA subtype, and HA-NA combined subtype. The reassortment rate being analysed in this study was classified into three levels: a) The overall reassortment rate for an internal segment (e.g. PB2) reported for HA subtype and for NA and for combined HA-NA was the overall mutation rate of HA,NA or combined HA-NA traits in substitutions per site per year; b) The rate of reassortment for an internal segment (e.g. PB2) starting from each different subtype (e.g. H1, N1, or H1N1) is the mean rate that averaged over the individual transition rates from certain subtype to all the other subtypes; c) The asymmetric rate of reassortment for an internal segment (e.g.PB2) starting from one subtype to another (e.g. H1 to H2, H2 to H1) was estimated from each relative transition rate multiplied by the corresponding clock rate per Monte Carlo Markov Chain (MCMC) sample.

For comparison, the reassortment rates were also estimated by MultiState in BayesTraits (version 1.0) [58], using a maximum likelihood method with 1000 bootstrap replications. Only the rates of HA and NA subtypes (not HA-NA joint subtype) could be estimated using Multistate, as only a maximum of 10 distinct states were allowed. The mean rates estimated by two different reconstruction methods (MCMC and ML) are similar (data not shown), although there was a significant shrinkage in uncertainty using the formal method (see Additional file 1: Figure S3). This result suggested that reassortment rates estimated with discrete trait models on the MCMC-based Bayesian framework were preferred.

### Robustness of random sampling

In order to generate datasets suitable for obtaining time-resolved trees, stratified subsampling was conducted using custom R scripts. Subtypes with more than 7 sequences were subjected to further analysis. In addition, to keep the pattern of the original phylogeny, sequences in isolated branches with extremely long branches were ignored. To retain a similar prevalence of isolates for each subtype, random subsampling was conducted to keep one sequence per subtype per host species per location per year, and was repeated 3 times (Additional file 2: Table S1, rows A1-A3). Among the three subgroups (A1, A2, and A3), the mean individual reassortment rate of each single transmission were similar to each other and with overlapping credible intervals (Cls) , which indicated that the estimated reassortment rates are robust by random sampling (Additional file 1: Figure S4). Thus, the reassortment rates shown in the results were the mean rate of the three subgroups.

### Test of sample size effect

The dataset in this study was obtained from the NCBI database, where the number of sequences is unevenly distributed among different subtypes. HPAI H5N1 AIV in particular accounts for a very high proportion of sequences due to large-scale surveillance efforts. To investigate a possible sample size effect on the reassortment rate, we estimated reassortment rates using two other subsampled datasets which comprised different numbers of representative sequences from the original datasets of 344. To identify whether the sample size of each subtypes would affect the reassortment rate, we further select representative sequences of different subtypes in two other different ways: 1) by reducing the number of the most over-represented subtype to equal the number of the rare subtype in the original dataset (Additional file 2: Table S1, row B); and 2) by reducing the number of sequences of all the subtypes to be the same as the rare subtype (Additional file 2: Table S1, row C). Representative isolates of the down-sampled datasets were selected according to the tree phylogeny of the original maximum likelihood tree generated with all the Eurasian AIV, using a software tool which searches for sequence clusters [59] (http://hiv.bio.ed.ac.uk/software.html), with 70% bootstrap support and a genetic distance lower than 0.045 specified. In this comparison, the reassortment rates among subtypes were similar in all the three datasets (Additional file 1: Figure S7). Thus, we can conclude that the sample size does not affect the reassortment pattern of the Eurasian AIV in our study.

### Estimation of evolutionary rates

The time scaled Bayesian phylogenies were generated with an uncorrelated log-normal relaxed molecular clock, and each branch was annotated with its mean substitution rate (ucld.mean). Additionally the nodes of the trees were annotated with the inferred HA, NA or HA-NA subtype states from the respective discrete trait models. The mean evolutionary rates per subtype were extracted from the trees, averaging over the rates on only those branches for which both the parent and child nodes were annotated with that subtype. The overall evolutionary rate of each segment was the ucld.mean rate of the MCMC tree phylogeny of each segment with 95% HPD intervals.

### Estimation of the dN/dS ratio

Selection of certain subtype on the tree phylogeny was analyzed with implementation of robust counting on the trees generated with Bayesian discrete models in Beast, which reconstructed the synonymous and non-synonymous change counts using a 3-partition codon model [60]. For segment 6 and 7, M1 and NS1 were analyzed instead of total length due to overlapping reading frames [61]. For either conditional (C) and unconditional (U) stochastic mapping realizations, the numbers of synonymous (S) substitutions *C*(S-C), *C*(S-U) and nonsynonymous (N) substitutions *C*(N-C), *C*(N-U) for each state on each branch were recorded, and then converted into d_N_/d_S_ values (indicated by certain state/subtype) by computing the ratio *C*(N-C)*/C*(S-C)*/C*(N-U)*/C*(S-U) [39]. The ratio per subtype was estimated from the ratio per branch across all the MCMC trees. The overall selective pressure of each segment was the mean difference between the mean number of total N and total S of across all the MCMC trees of certain segment with 95%HPD intervals.

We also used Single Likelihood Ancestor Counting (SLAC) in HyPhy (http://www.datamonkey.org/) to estimate dn/ds per subtype. Here we used the original datasets (1185 AIV in total) which have more sequences of each subtype than the subsampling dataset. The HKY85 nucleotide substitution model was used in SLAC-based analyses.

## Additional files

Models, priors and settings used in this analysis are provided in the XML used as input for BEAST 1.7.3, which are available from the Dryad Digital Repository: http://doi.org/10.5061/dryad.246pr.

## Competing interests

The authors declare that no competing interests exist.

## Authors’ contributions

LL performed the phylogenetic and discrete trait mapping analyses, was involved in the study design and drafted the manuscript. SJL provided the major scripts for R programs used in this study, helped with the study design and provided guidance on the analysis. AJLB conceived the study, provided guidance on its design and drafted the manuscript. All authors read and approved the final manuscript.

## Supplementary Material

Additional file 1 **Figure S1.** Maximum likelihood tree of 2996 AIV of PB2 segment. **Figure S2.** Bayesian maximum clade credibility phylogenies for the five internal segments (A: HA subtype, B: NA subtype, C: Combined HA-NA subtype) of Eurasian Avian influenza viruses. **Figure S3.** Distribution of variance of reassortment rate using BSSVS and Bayes Traits. **Figure S4.** Robustness of reassortment rate (HA, NA and HA-NA combined subtype) estimates using BSSVS among 3 random sampling subgroups. **Figure S5.** Heat map of reassortment rates of remain 5 internal segments (PB1, PA, NP, M, NS) of different subtypes (HA, NA and both HA and NA). **Figure S6.** Distribution of reassortment rates in the PB2 heat maps for H, N, and H-N subtypes in rows (blue) and columns (red) (Figure 2 and Additional file 2: Table S2). **Figure S7.** Heat map of reassortment rates of HA, NA and combined HA-NA subtypes of PB2 segment on three AIV datasets of different sample sizes (Group A, B and C are correspondent to Additional file 2: Table S1). **Figure S8.** Correlation between proportion of AIV in wild birds and reassortment rate of different subtypes. **Figure S9.** Correlation between proportion of AIV in Anseriformes and reassortment rate of different subtypes. **Figure S10.** Correlation between the proportion of AIV in wild anseriformes and the reassortment rate of different subtypes. **Figure S11.** Correlation between proportion of AIV in domestic anseriformes and reassortment rate of different subtypes. **Figure S12.** Correlation between proportion of AIV in domestic galliformes and reassortment rate per subtype. **Figure S13.** Correlation between evolutionary rate and reassortment rate per subtype. **Figure S14.** Correlation between inter-subtype reassortment rate and dN/dS (using SLAC). Click here for file

Additional file 2 **Table S1.** Number of sequences by subtype in each subgroup. **Table S2.** Matrix of reassortment rate between pairs of subtypes (HA, NA and HA-NA combined) of 6 internal segments. **Table S3.** Partial correlation between reassortment and domestic proportion or dN/dS ratio. **Table S4.** Number of selected sites for 13 subtypes and the correlation with inter-subtype reassortment rate in each internal segment. **Table S5.** Sequences for the discrete traits analysis. Information for subsampling data set A2 (n = 344, Additional file 2: Table S1) of Eurasian AIV sequences used in this discrete traits analysis. Isolate name, subtype, host species, date of collection and the estimated decimal date are listed. Click here for file
