# Supplementary material for: Reassortment patterns of avian influenza virus internal segments among different subtypes
Source: BMC Evol Biol. 2014 Jan 24;14:16. doi: 10.1186/1471-2148-14-16 (PMC3905155; doi:10.1186/1471-2148-14-16)
Supplement: Additional file 1 — Figure S1. Maximum likelihood tree of 2996 AIV of PB2 segment. Figure S2. Bayesian maximum clade credibility phylogenies for the five internal segments (A: HA subtype, B: NA subtype, C: Combined HA-NA subtype) of Eurasian Avian influenza viruses. Figure S3. Distribution of variance of reassortment rate using BSSVS and Bayes Traits. Figure S4. Robustness of reassortment rate (HA, NA and HA-NA combined subtype) estimates using BSSVS among 3 random sampling subgroups. Figure S5. Heat map of reassortment rates of remain 5 internal segments (PB1, PA, NP, M, NS) of different subtypes (HA, NA and both HA and NA). Figure S6. Distribution of reassortment rates in the PB2 heat maps for H, N, and H-N subtypes in rows (blue) and columns (red) (Figure 2 and Additional file 2: Table S2). Figure S7. Heat map of reassortment rates of HA, NA and combined HA-NA subtypes of PB2 segment on three AIV datasets of different sample sizes (Group A, B and C are correspondent to Additional file 2: Table S1). Figure S8. Correlation between proportion of AIV in wild birds and reassortment rate of different subtypes. Figure S9. Correlation between proportion of AIV in Anseriformes and reassortment rate of different subtypes. Figure S10. Correlation between the proportion of AIV in wild anseriformes and the reassortment rate of different subtypes. Figure S11. Correlation between proportion of AIV in domestic anseriformes and reassortment rate of different subtypes. Figure S12. Correlation between proportion of AIV in domestic galliformes and reassortment rate per subtype. Figure S13. Correlation between evolutionary rate and reassortment rate per subtype. Figure S14. Correlation between inter-subtype reassortment rate and dN/dS (using SLAC). [file 1471-2148-14-16-S1.pdf]

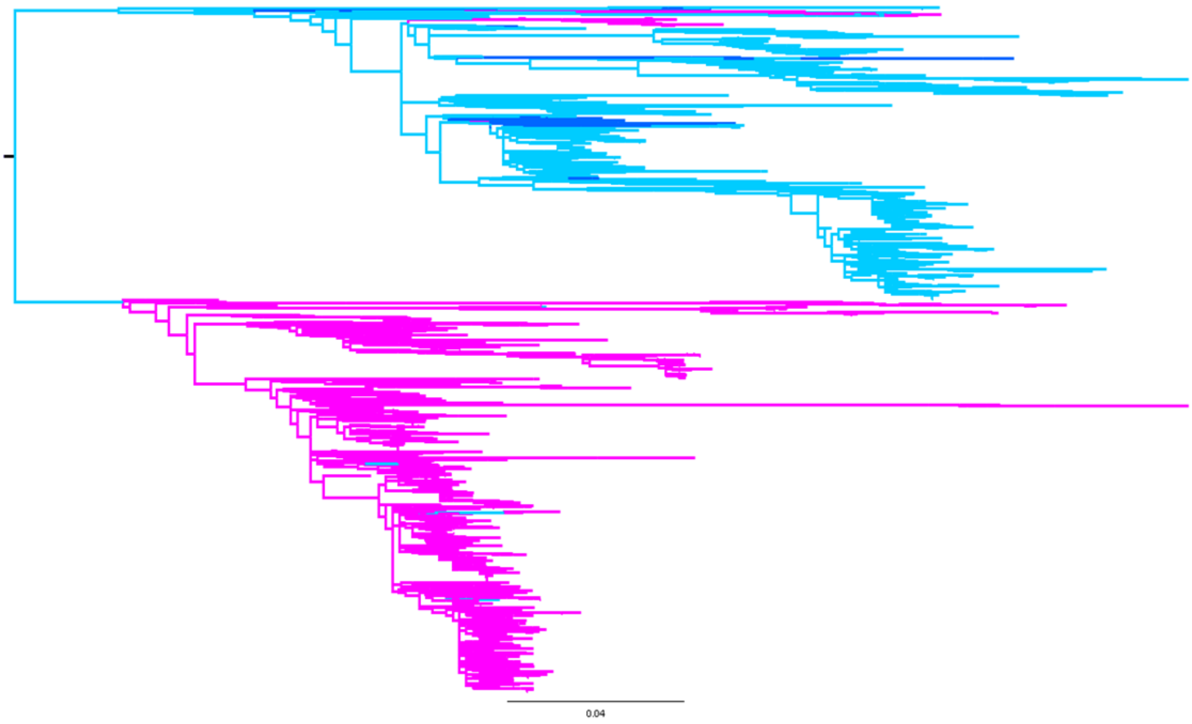

Figure S1

Maximum likelihood tree of 2996 AIV of PB2 segment. AIV from Eurasian region are coloured by blue (those from Oceania are coloured by dark blue), and those from the American regions are coloured by pink.

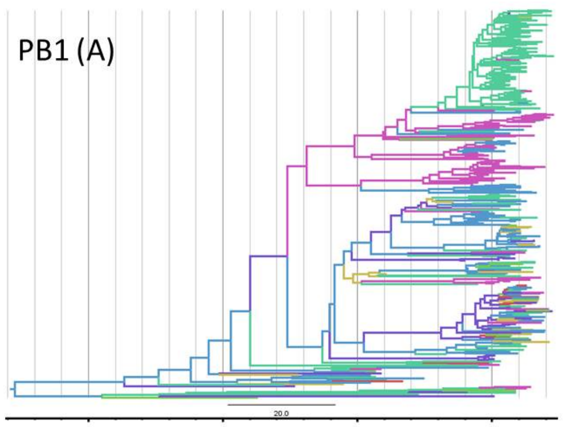
PB1 (A)
1925.0
1950.0
1975.0
2000.0

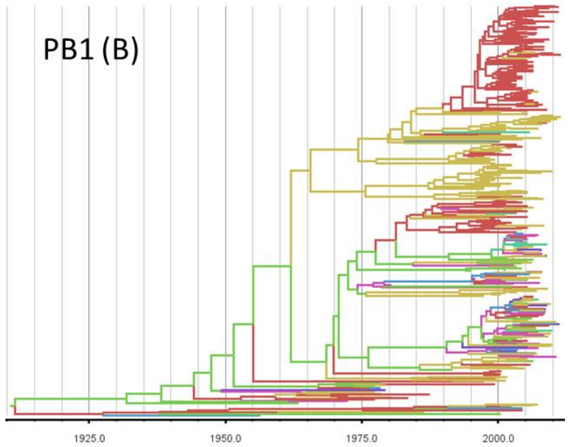
PB1 (B)
1925.0
1950.0
1975.0
2000.0

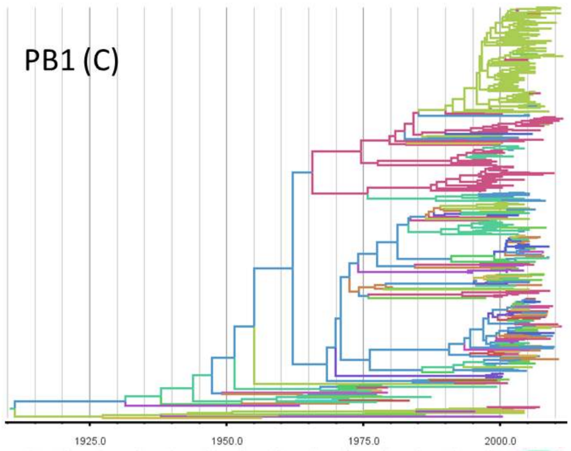
PB1 (C)
1925.0
1950.0
1975.0
2000.0

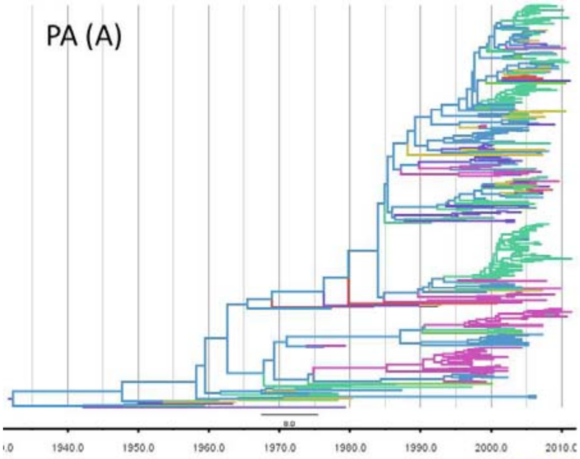
PA (A)
1940.0
1950.0
1960.0
1970.0
1980.0
1990.0
2000.0

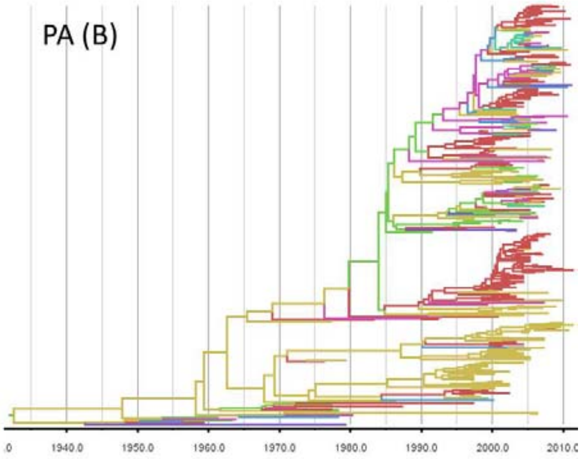
PA (B)
1940.0
1950.0
1960.0
1970.0
1980.0
1990.0
2000.0

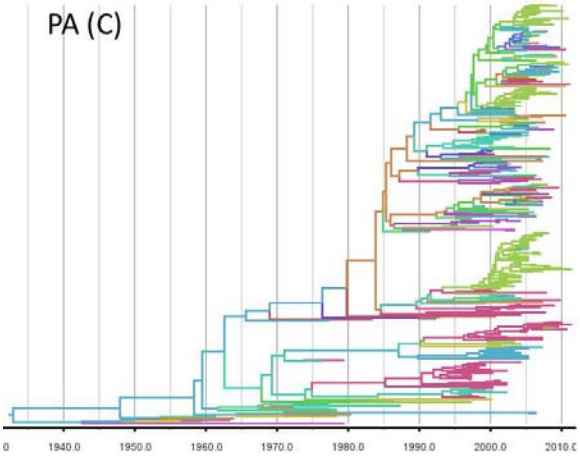
PA (C)
1940.0
1950.0
1960.0
1970.0
1980.0
1990.0
2000.0

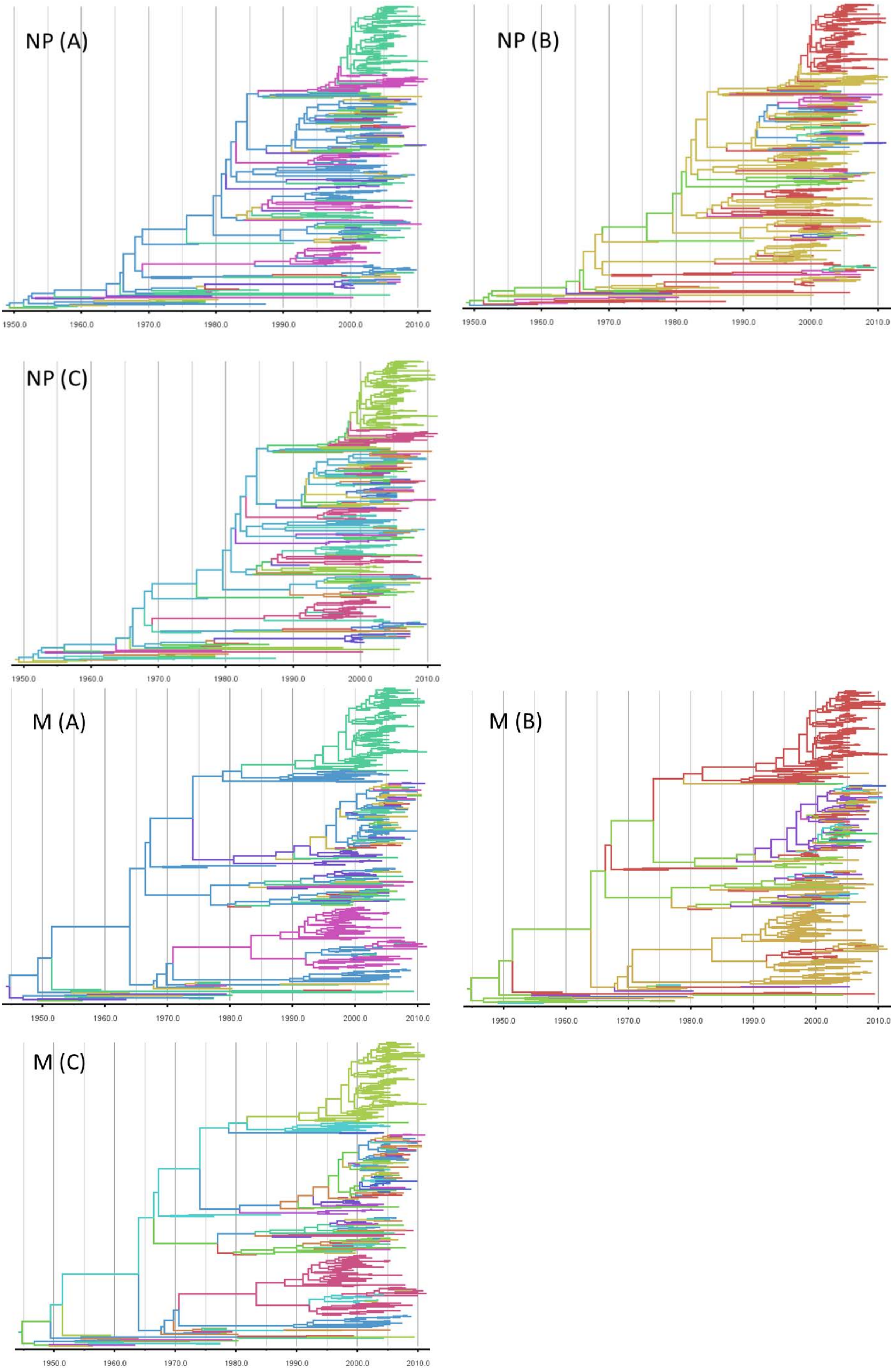

NP (A)
1950.0
1960.0
1970.0
1980.0
1990.0
2000.0
2010.0
NP (B)
1950.0
1960.0
1970.0
1980.0
1990.0
2000.0
2010.0
NP (C)
1950.0
1960.0
1970.0
1980.0
1990.0
2000.0
2010.0
M (A)
1950.0
1960.0
1970.0
1980.0
1990.0
2000.0
2010.0
M (B)
1950.0
1960.0
1970.0
1980.0
1990.0
2000.0
2010.0
M (C)
1950.0
1960.0
1970.0
1980.0
1990.0
2000.0
2010.0

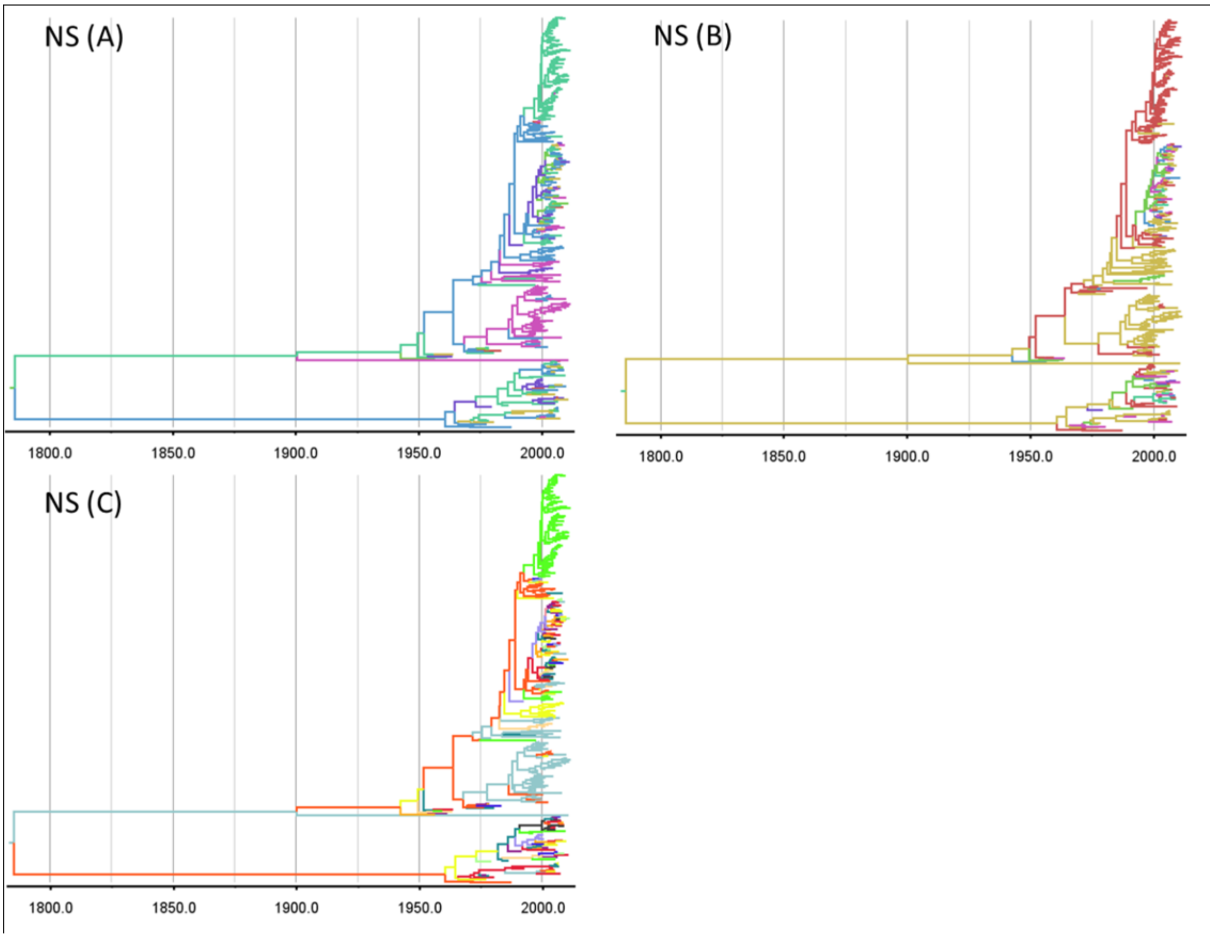

Figure S2

Bayesian maximum clade credibility phylogenies for the five internal segments (A: HA subtype, B: NA subtype, C: Combined HA-NA subtype) of Eurasian Avian influenza viruses. Branches were coloured according to the different subtypes of their descendent nodes. The colour markers are the same as Figure 2.

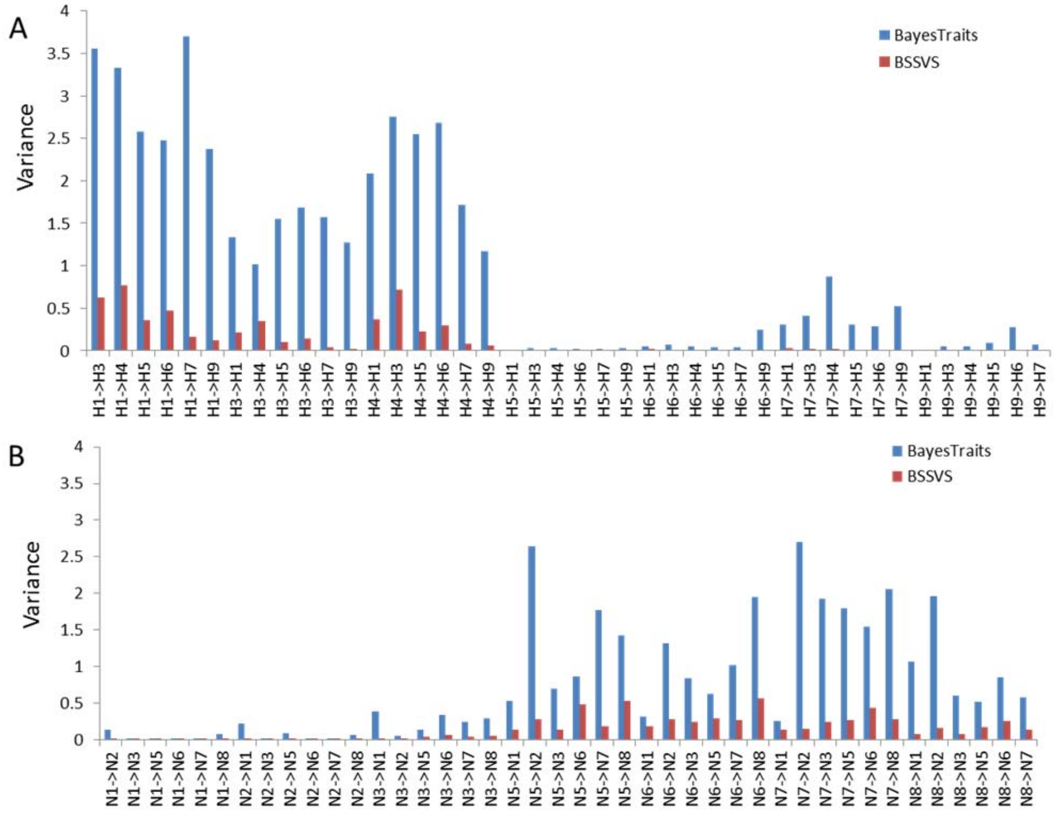

Figure S3

Distribution of variance of reassortment rate using BSSVS and Bayes Traits. PB2 segment of HA subtype (A) and NA subtype (B). Variance of estimated reassortment rate between pairs of subtypes using BayesTraits (blue); Variance of estimated reassortment rate between pairs of subtypes using BSSVS (red).

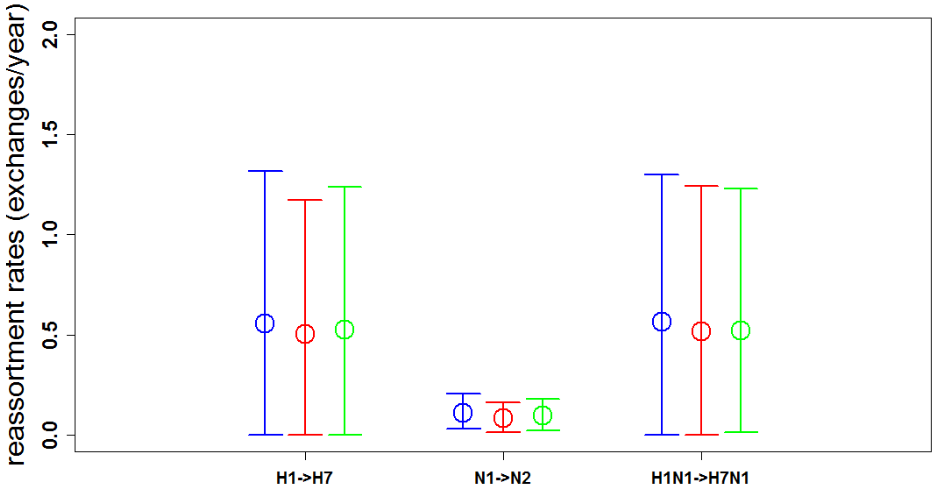

Figure S4

Robustness of reassortment rate (HA, NA and HA-NA combined subtype) estimates using BSSVS among 3 random sampling subgroups. As for clarity, only one reassortment rate per HA,NA and HA-NA combined subtype for PB2 segment was shown respectively, with mean reassortment rates and 95% Highest probability density (HPD) intervals. Blue: group1; Red: group 2; Green: group 3.The complete rate of each transition were shown in Table S2.

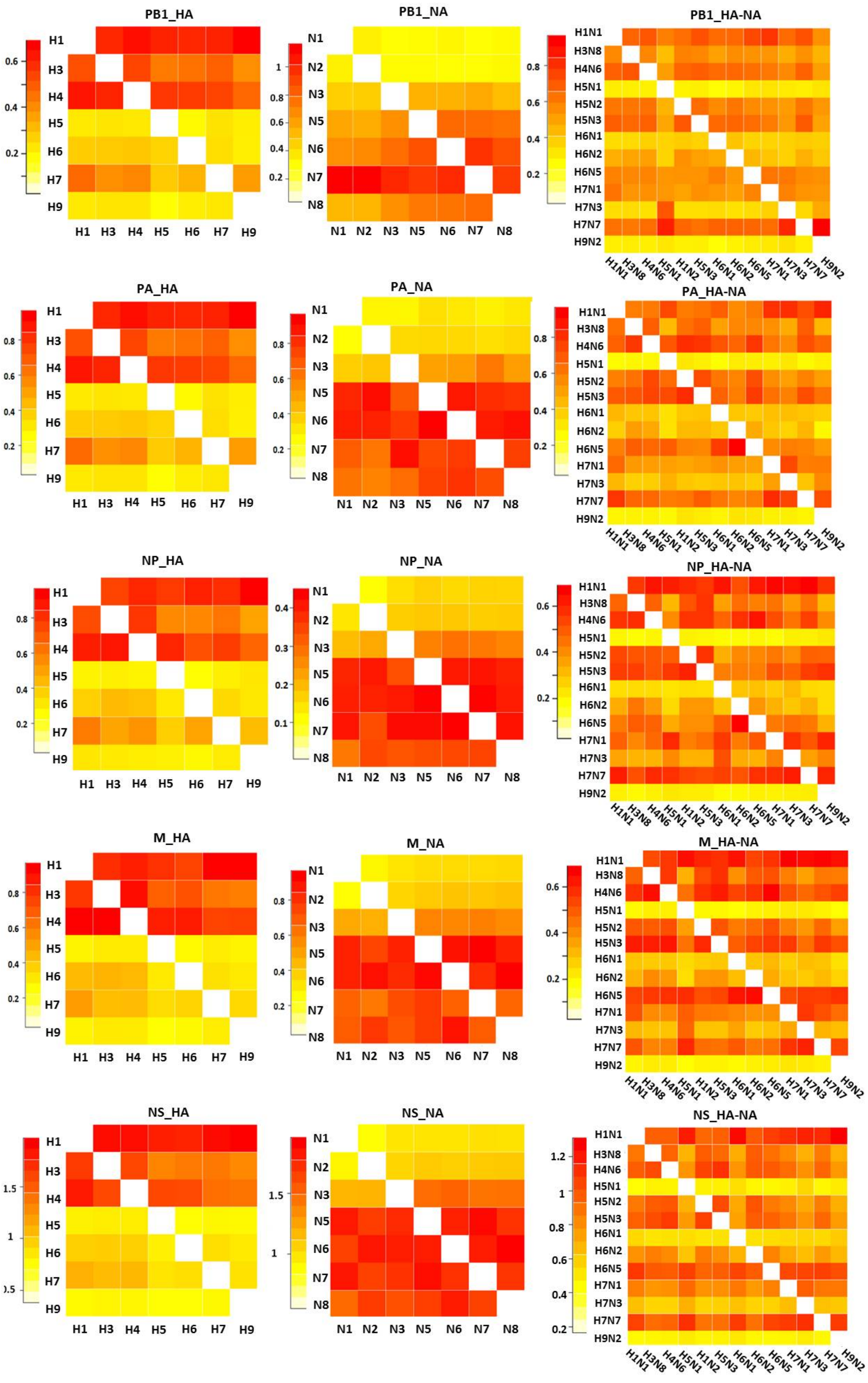
PB1_HA
PB1_NA
PB1_HA-NA
PA_HA
PA_NA
PA_HA-NA
NP_HA
NP_NA
NP_HA-NA
M_HA
M_NA
M_HA-NA
NS_HA
NS_NA
NS_HA-NA
H1 H3 H4 H5 H6 H7 H9
N1 N2 N3 N5 N6 N7 N8
H1N1 H3N8 H4N6 H5N1 H5N2 H5N3 H6N1 H6N2 H6N5 H7N1 H7N3 H7N7 H9N2

Figure S5

Heat map of reassortment rates of remain 5 internal segments (PB1, PA, NP, M, NS) of different subtypes (HA, NA and both HA and NA). Subtypes labelled on left side are as donors, subtypes labelled in the bottom are as recipients. The values of rates from low to high are represented colors from yellow to red. The scale of rates is on the left hand side of each plot.

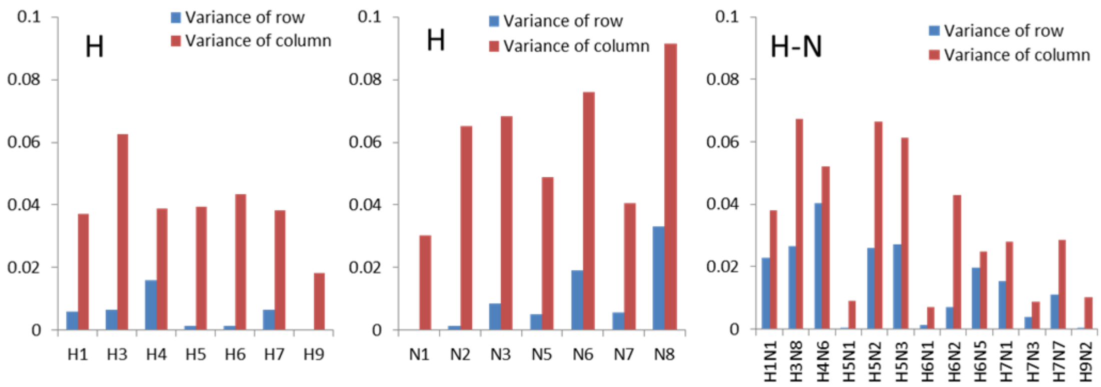

Figure S6

Distribution of reassortment rates in the PB2 heat maps for H, N, and H-N subtypes in rows (blue) and columns (red) (Fig. 2 and Table S2). The variance of the reassortment rate in the rows (blue) is lower than that in the columns (red); indicating the asymmetry of the reassortment rates. Reassortment rates from H and N subtypes as donors (rows) to other subtypes are less variable than rates from all subtypes to H and N subtypes as recipients (columns).

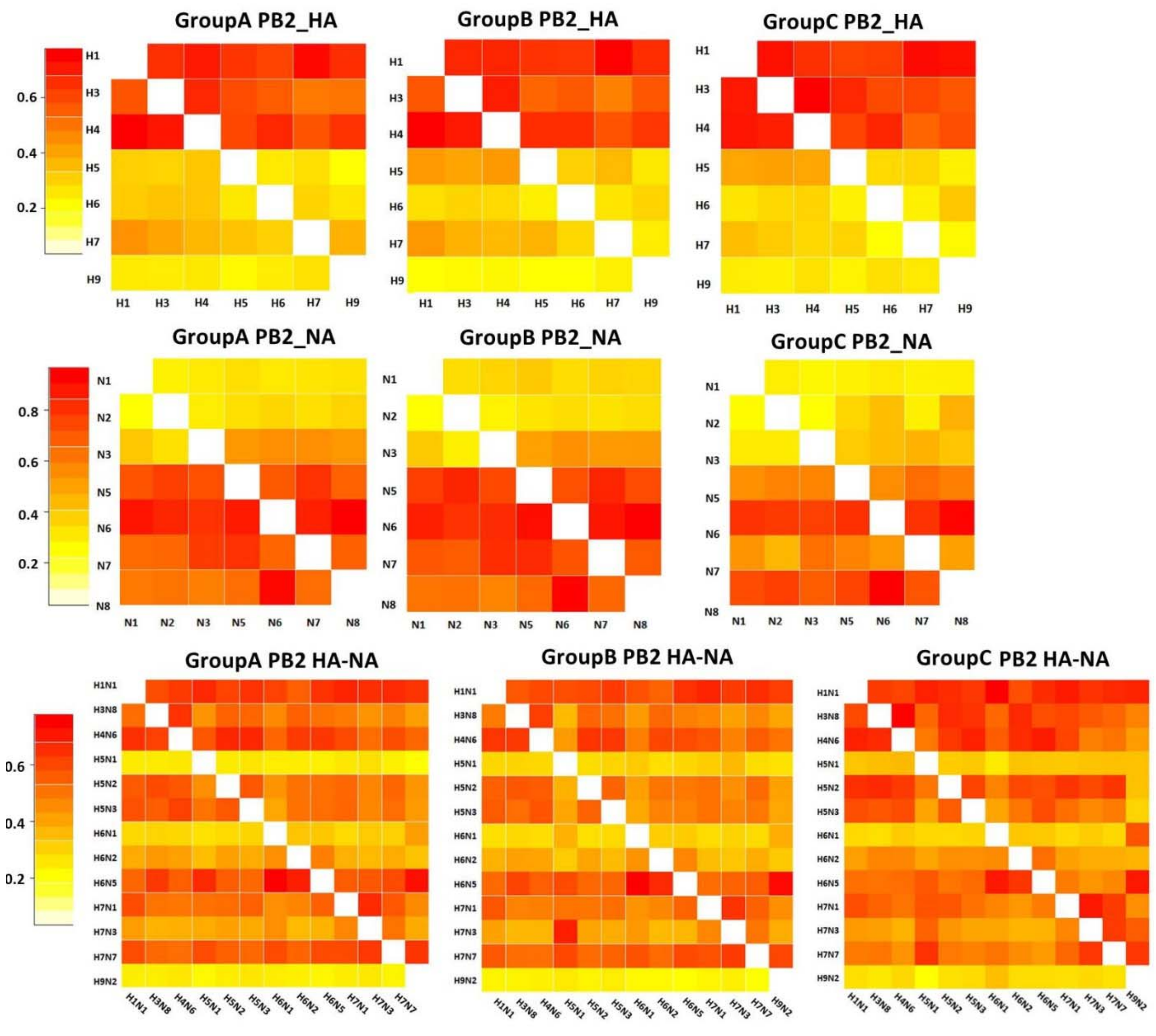

Figure S7

Heat map of reassortment rates of HA, NA and combined HA-NA subtypes of PB2 segment on three AIV datasets of different sample sizes (Group A, B and C are correspondent to tableS1). Subtypes labelled on left side are as donors, subtypes labelled in the bottom are as recipients. The values of rates from low to high are represented colors from yellow to red. The scale of rates is on the left hand side of each plot.

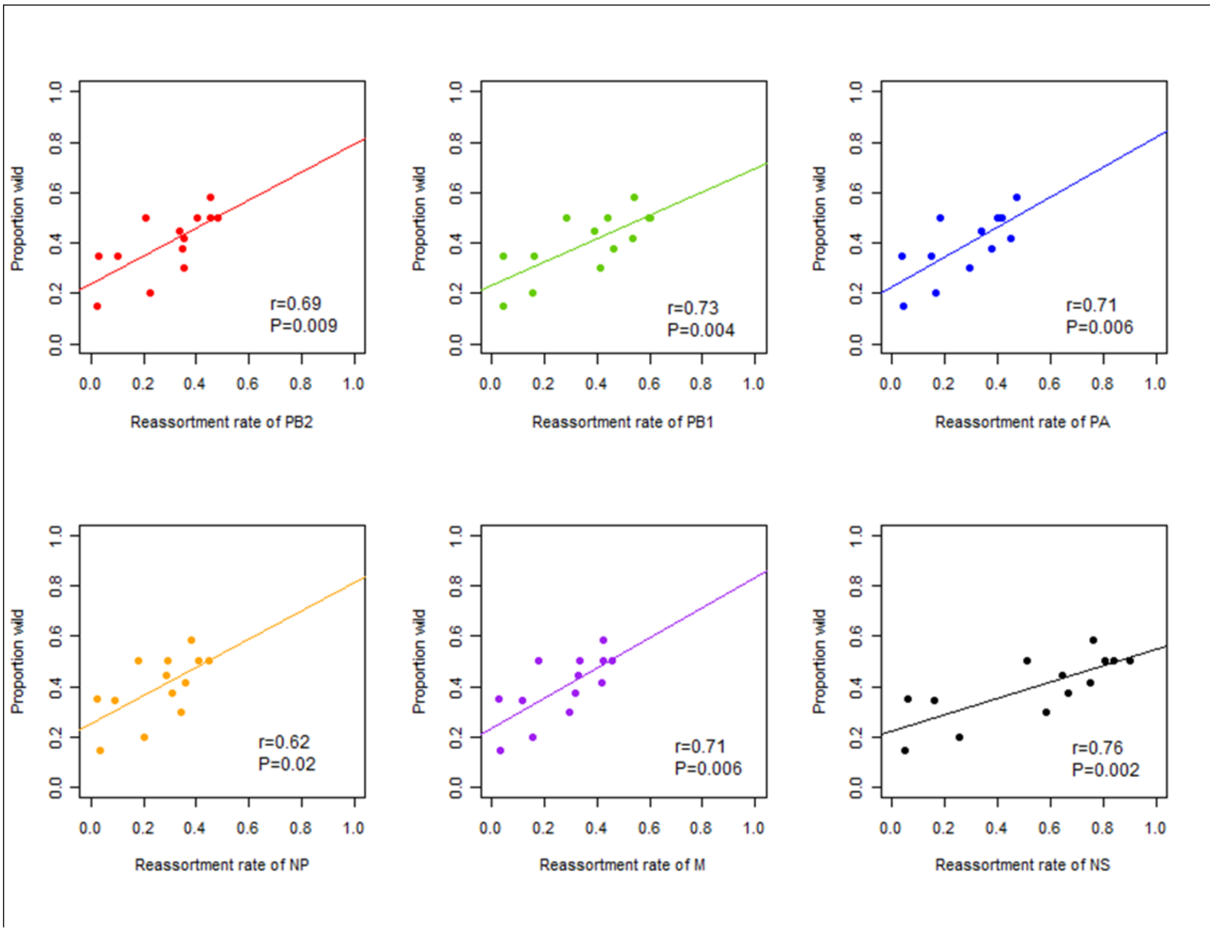

.

Figure S8

Correlation between proportion of AIV in wild birds and reassortment rate of different subtypes. The scatterplot of reassortment rate of HA-NA combined subtype against proportion of wild birds per subtype were represented for 6 internal segments by different colors: PB2 (red); PB1 (green); PA (blue); NP (orange); M1 (purple); NS1 (black).

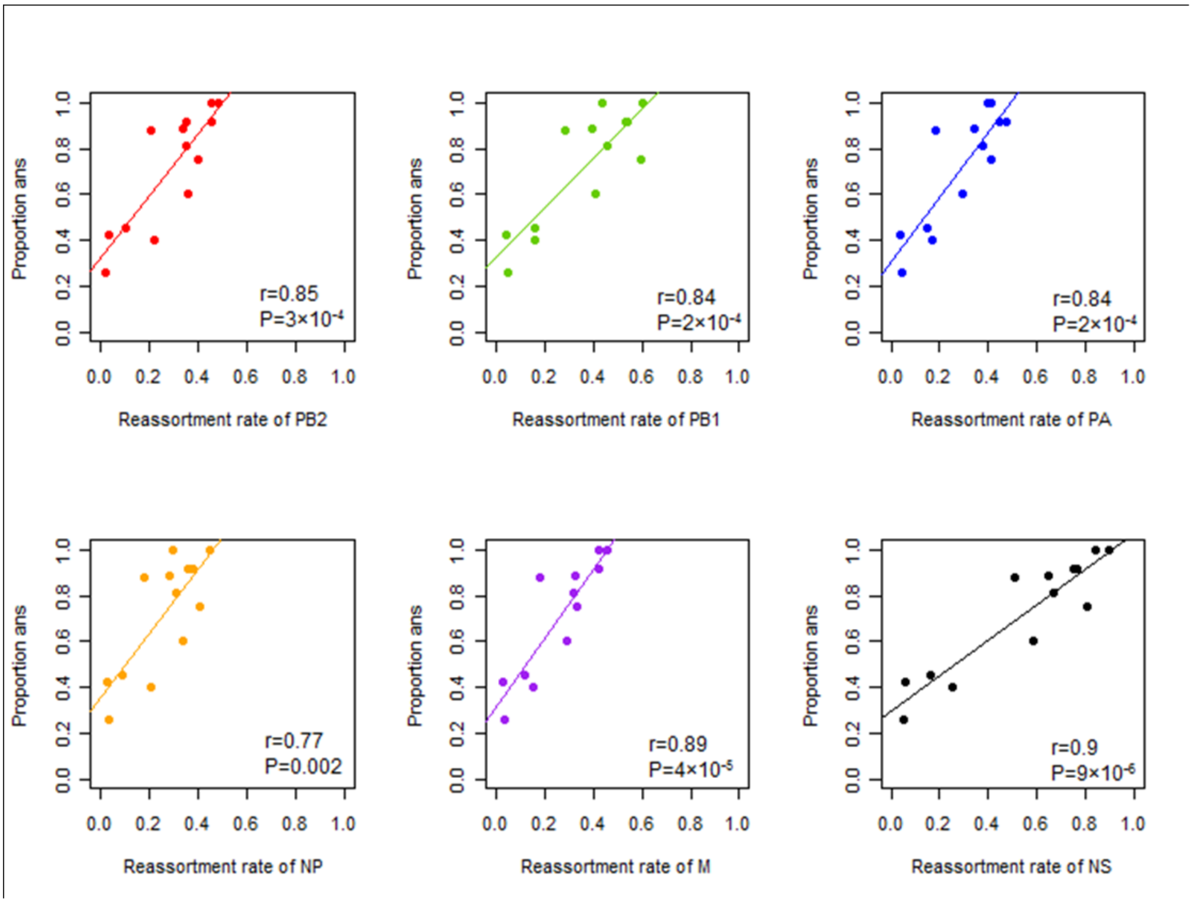

Figure S9

Correlation between proportion of AIV in anseriformes and reassortment rate of different subtypes. The scatterplot of reassortment rate of HA-NA combined subtype against proportion of domestic birds of anseriformes per subtype were represented for 6 internal segments by different colors: PB2 (red); PB1 (green); PA (blue); NP (orange); M1 (purple); NS1 (black).

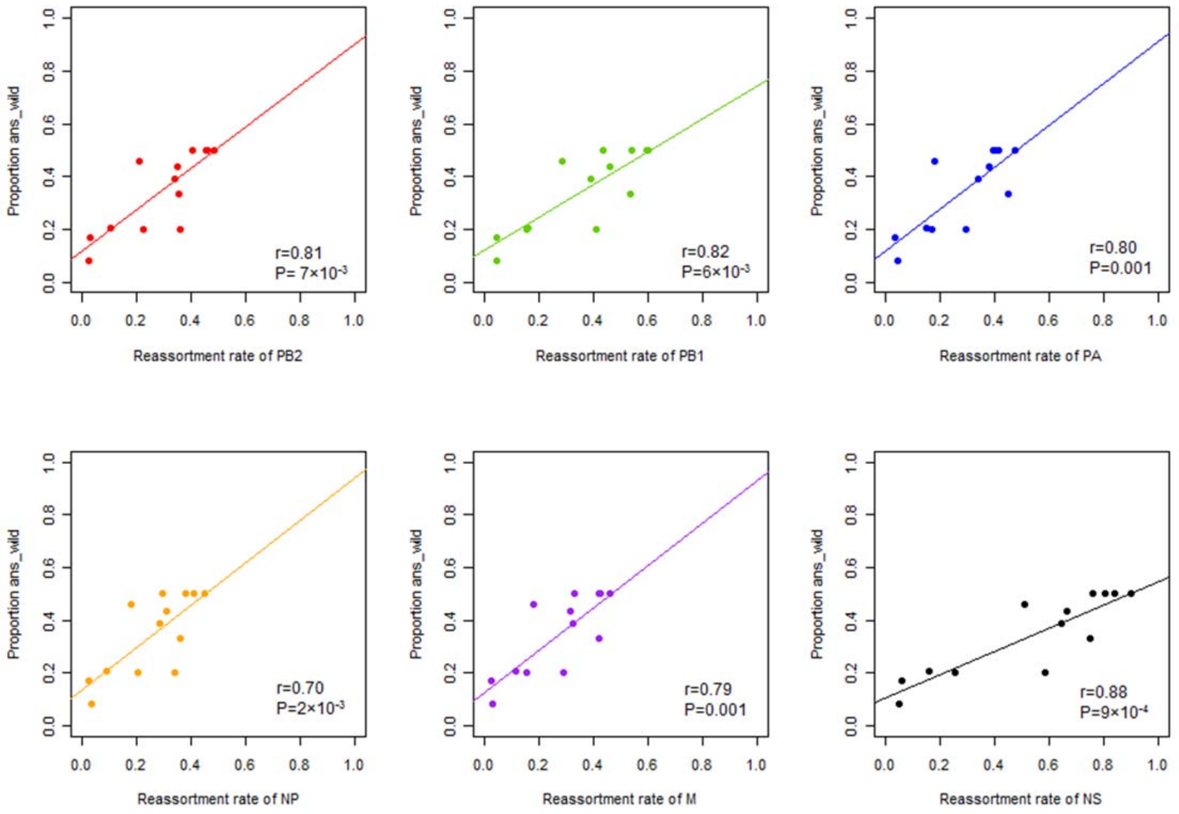

Figure S10

Correlation between the proportion of AIV in wild anseriformes and the reassortment rate of different subtypes. The scatterplot of reassortment rate of HA-NA combined subtype against proportion of wild anseriformes per subtype were represented for 6 internal segments by different colors: PB2 (red); PB1 (green); PA (blue); NP (orange); M1 (purple); NS1 (black).

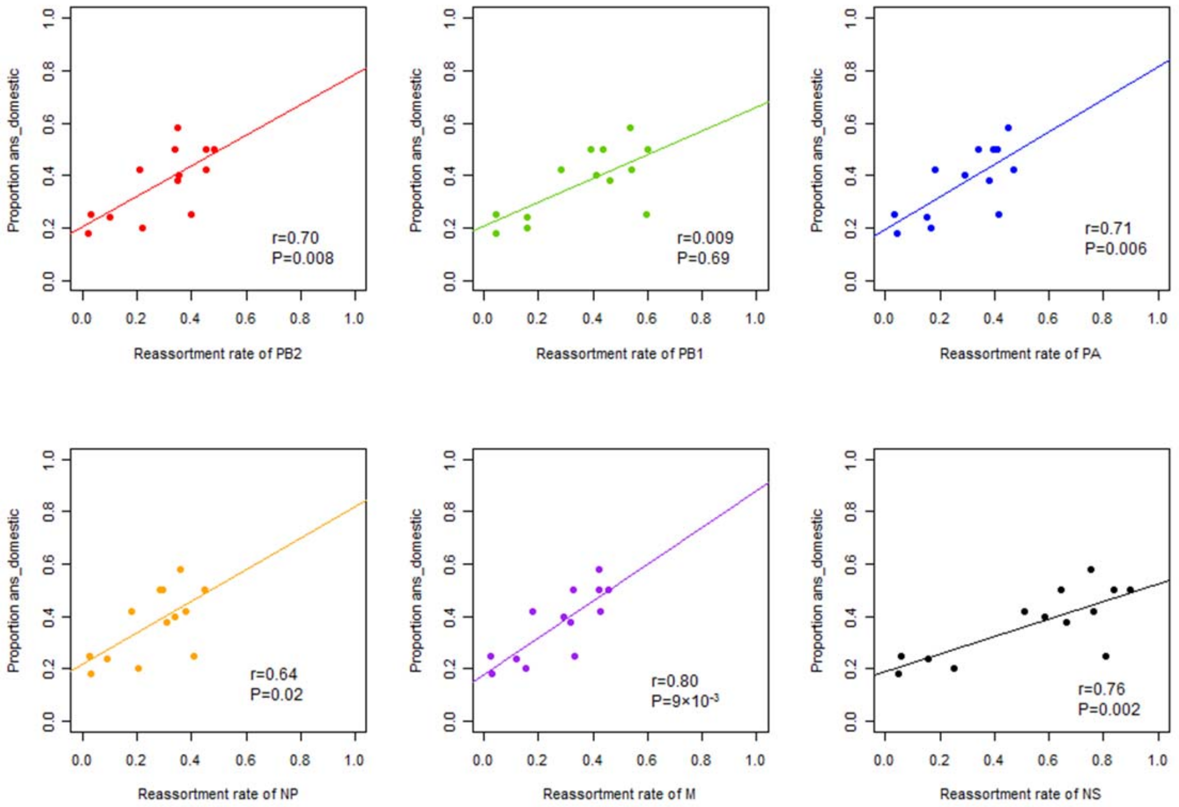

Figure S11

Correlation between proportion of AIV in domestic anseriformes and reassortment rate of different subtypes. The scatterplot of reassortment rate of HA-NA combined subtype against proportion of domestic anseriformes per subtype were represented for 6 internal segments by different colors: PB2 (red); PB1 (green); PA (blue); NP (orange); M1 (purple); NS1 (black).

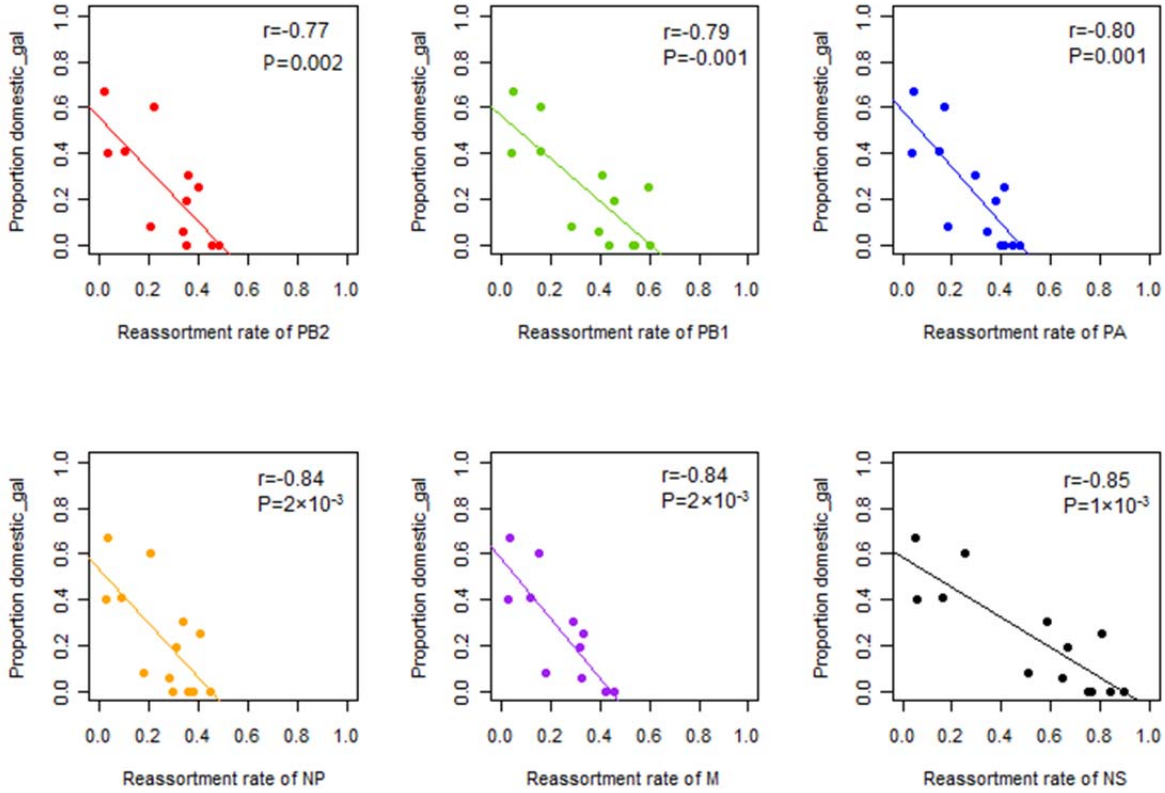

Figure S12

Correlation between proportion of AIV in domestic galliformes and reassortment rate per subtype. The scatterplot of reassortment rate of HA-NA combined subtype against proportion of domestic galliformes per subtype were represented for 6 internal segments by different colors: PB2 (red); PB1 (green); PA (blue); NP (orange); M1 (purple); NS1 (black).

Figure. S13

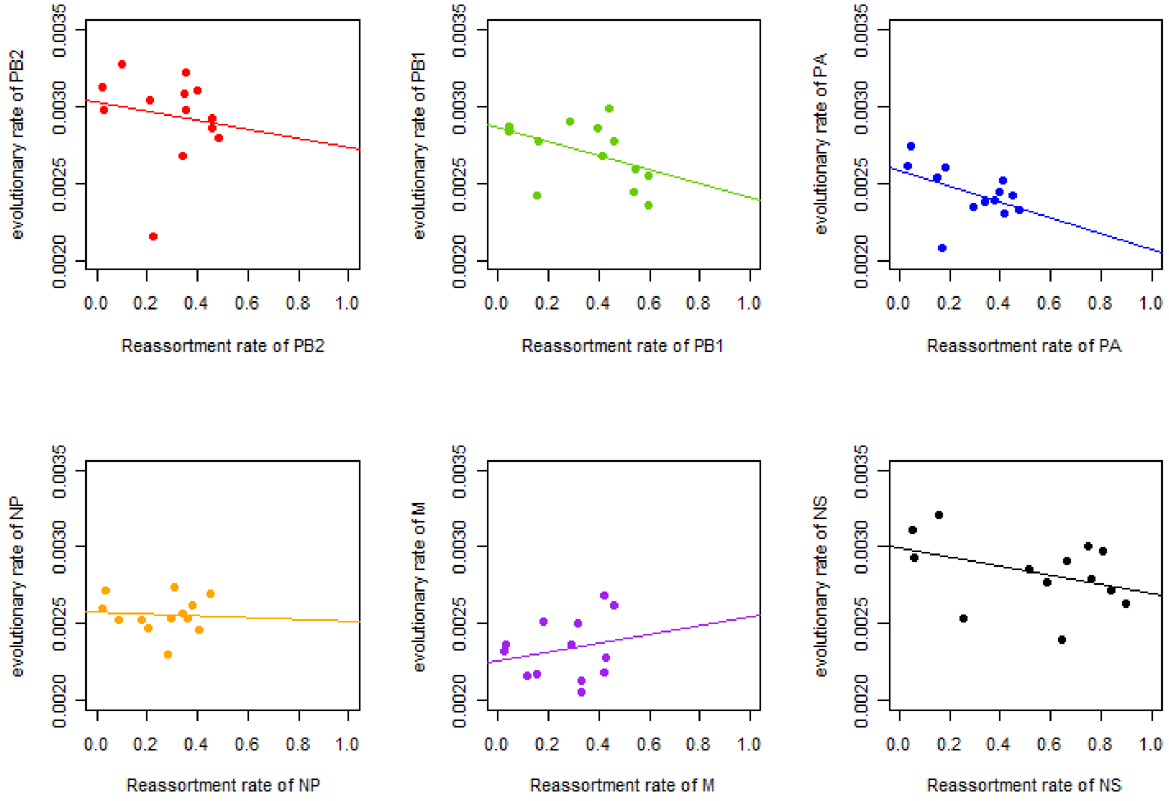

Figure. S13: Correlation between evolutionary rate and reassortment rate per subtype. The scatterplot of reassortment rate of HA-NA combined subtype against relaxed clock rate per subtype were represented for 6 internal segments by different colors: PB2 (red); PB1 (green); PA (blue); NP (orange); M1 (purple); NS1 (black).

Figure. S14

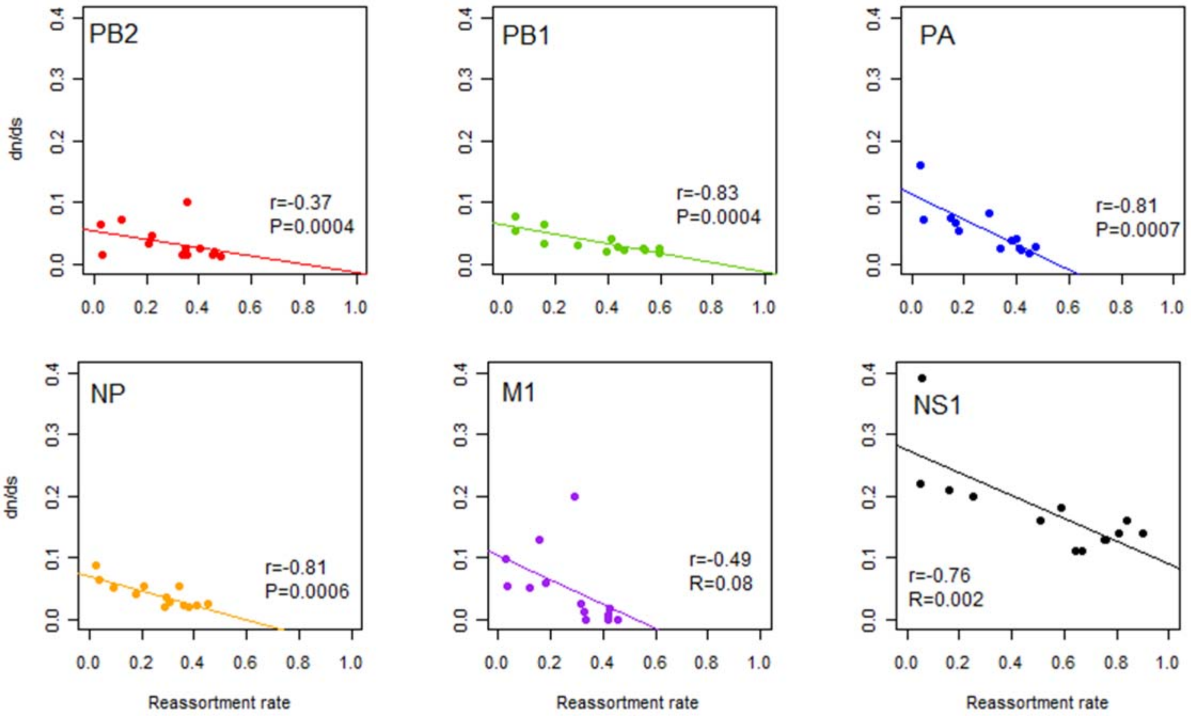

Figure S14 Correlation between inter-subtype reassortment rate and dN/dS (using SLAC). The scatterplot of reassortment rate of HA-NA combined subtype against dN/dS ratio of 6 internal segments were represented by different colors: PB2 (red); PB1 (green); PA (blue); NP (orange); M1 (purple); NS1 (black).
