# Supplementary material for: Reassortment patterns of avian influenza virus internal segments among different subtypes
Source: BMC Evol Biol. 2014 Jan 24;14:16. doi: 10.1186/1471-2148-14-16 (PMC3905155; doi:10.1186/1471-2148-14-16)
Supplement: Additional file 2 — Table S1. Number of sequences by subtype in each subgroup. Table S2. Matrix of reassortment rate between pairs of subtypes (HA, NA and HA-NA combined) of 6 internal segments. Table S3. Partial correlation between reassortment and domestic proportion or dN/dS ratio. Table S4. Number of selected sites for 13 subtypes and the correlation with inter-subtype reassortment rate in each internal segment. Table S5. Sequences for the discrete traits analysis. Information for subsampling data set A2 (n = 344, Additional file 2: Table S1) of Eurasian AIV sequences used in this discrete traits analysis. Isolate name, subtype, host species, date of collection and the estimated decimal date are listed. [file 1471-2148-14-16-S2.pdf]

Table S1 Number of sequences by subtype in each subgroup

Group	H1N1	H3N8	H4N6	H5N1	H5N2	H5N3	H6N1	H6N2	H6N5	H7N1	H7N3	H7N7	H9N2	SUM
A1	7	19	12	100	17	12	31	36	10	10	12	9	68	344
A2	7	18	12	129	16	12	29	24	8	10	10	8	61	344
A3	7	19	12	126	17	12	30	23	8	10	10	8	61	344
B	7	18	12	10	16	12	29	24	8	10	10	8	61	225
C	7	10	10	10	10	10	10	10	10	10	10	9	10	127

Table S2 Matrix of reassortment rate between pairs of subtypes (HA, NA and HA-NA combined) of 6 internal segments. Reassortment rates in this table are represented as heat map in figure 2. Both forward and reverse rates are shown, with subtypes labeled on the left side acting as donors and subtypes labeled at the bottom acting as recipients. The values of the mean reassortment rates, in exchanges per year. The transition rates with BF<3 (the estimated indicator for HA/NA subtype < 0.3, for HA-NA subtype< 0.2) are labelled in grey.

HA							
PB2	H1	H3	H4	H5	H6	H7	H9
H1		0.55	0.51	0.46	0.45	0.50	0.33
H3	0.34		0.42	0.42	0.40	0.26	0.24
H4	0.53	0.63		0.39	0.46	0.32	0.30
H5	0.08	0.09	0.11		0.04	0.03	0.02
H6	0.08	0.15	0.10	0.05		0.05	0.08
H7	0.22	0.28	0.16	0.16	0.11		0.05
H9	0.02	0.02	0.02	0.01	0.03	0.02	
PB1	H1	H3	H4	H5	H6	H7	H9
H1		0.63	0.62	0.40	0.68	0.59	0.30
H3	0.35		0.47	0.25	0.45	0.42	0.14
H4	0.46	0.64		0.37	0.62	0.50	0.19
H5	0.05	0.04	0.04		0.02	0.05	0.03
H6	0.11	0.13	0.13	0.10		0.07	0.03
H7	0.30	0.31	0.31	0.12	0.24		0.12
H9	0.05	0.05	0.05	0.03	0.06	0.04	
PA	H1	H3	H4	H5	H6	H7	H9
H1		0.40	0.39	0.28	0.50	0.37	0.41
H3	0.28		0.56	0.26	0.51	0.30	0.23
H4	0.32	0.83		0.56	0.81	0.36	0.28
H5	0.04	0.05	0.06		0.05	0.04	0.03
H6	0.06	0.20	0.23	0.09		0.05	0.04
H7	0.14	0.17	0.17	0.07	0.19		0.08
H9	0.04	0.04	0.04	0.03	0.05	0.03	
NP	H1	H3	H4	H5	H6	H7	H9
H1		0.46	0.49	0.38	0.52	0.44	0.42
H3	0.31		0.45	0.23	0.29	0.28	0.14
H4	0.40	0.58		0.42	0.42	0.40	0.25
H5	0.02	0.02	0.03		0.02	0.03	0.02
H6	0.08	0.16	0.14	0.05		0.07	0.02
H7	0.23	0.21	0.24	0.10	0.23		0.10
H9	0.05	0.04	0.04	0.04	0.03	0.03	
M	H1	H3	H4	H5	H6	H7	H9
H1		0.71	0.69	0.42	0.62	0.49	0.31
H3	0.38		0.75	0.33	0.58	0.26	0.16
H4	0.48	0.88		0.46	0.76	0.36	0.24
H5	0.03	0.03	0.03		0.03	0.03	0.02
H6	0.13	0.22	0.19	0.06		0.05	0.03
H7	0.19	0.19	0.18	0.09	0.15		0.05
H9	0.03	0.03	0.03	0.02	0.03	0.02	
NS	H1	H3	H4	H5	H6	H7	H9
H1		1.62	1.50	0.81	1.28	0.98	0.69
H3	0.92		1.14	0.46	0.79	0.55	0.32
H4	1.06	1.20		0.67	1.06	0.58	0.38
H5	0.09	0.09	0.09		0.05	0.04	0.03
H6	0.24	0.31	0.28	0.08		0.14	0.06
H7	0.38	0.43	0.43	0.13	0.23		0.09
H9	0.05	0.04	0.04	0.03	0.04	0.04	

NA							
PB2	N1	N2	N3	N5	N6	N7	N8
N1		0.08	0.04	0.07	0.06	0.05	0.08
N2	0.05		0.03	0.06	0.13	0.06	0.13

N3	0.18	0.14		0.27	0.39	0.27	0.37
N5	0.44	0.67	0.56		0.58	0.52	0.58
N6	0.58	0.76	0.66	0.63		0.56	0.97
N7	0.39	0.54	0.63	0.57	0.53		0.59
N8	0.36	0.50	0.40	0.39	0.87	0.36	
PB1	N1	N2	N3	N5	N6	N7	N8
N1		0.06	0.05	0.04	0.09	0.09	0.06
N2	0.07		0.06	0.04	0.06	0.10	0.06
N3	0.18	0.22		0.25	0.35	0.36	0.31
N5	0.30	0.37	0.41		0.62	0.56	0.64
N6	0.38	0.53	0.53	0.53		0.74	0.79
N7	0.82	1.15	0.76	0.59	0.86		0.87
N8	0.25	0.33	0.41	0.41	0.58	0.56	
PA	N1	N2	N3	N5	N6	N7	N8
N1		0.09	0.04	0.08	0.08	0.06	0.07
N2	0.06		0.10	0.11	0.14	0.10	0.13
N3	0.17	0.23		0.30	0.35	0.30	0.36
N5	0.57	0.82	0.38		0.81	0.45	0.79
N6	0.59	0.76	0.45	0.89		0.49	0.93
N7	0.45	0.48	0.55	0.63	0.69		0.74
N8	0.39	0.44	0.34	0.66	0.72	0.39	
NP	N1	N2	N3	N5	N6	N7	N8
N1		0.05	0.03	0.04	0.04	0.04	0.06
N2	0.09		0.06	0.06	0.08	0.04	0.07
N3	0.12	0.20		0.19	0.26	0.18	0.22
N5	0.25	0.45	0.23		0.44	0.34	0.42
N6	0.26	0.44	0.31	0.43		0.37	0.44
N7	0.26	0.36	0.33	0.42	0.50		0.46
N8	0.18	0.37	0.22	0.29	0.34	0.26	
M	N1	N2	N3	N5	N6	N7	N8
N1		0.04	0.04	0.03	0.03	0.03	0.03
N2	0.04		0.43	0.69	0.09	0.06	0.11
N3	0.13	0.15		0.25	0.31	0.22	0.31
N5	0.42	0.52	0.42		0.76	0.36	0.78
N6	0.45	0.58	0.43	0.69		0.58	0.93
N7	0.26	0.27	0.35	0.40	0.44		0.43
N8	0.32	0.46	0.34	0.54	0.71	0.25	
NS	N1	N2	N3	N5	N6	N7	N8
N1		0.08	0.04	0.05	0.04	0.04	0.05
N2	0.05		0.13	0.16	0.17	0.13	0.20
N3	0.22	0.36		0.57	0.77	0.48	0.75
N5	0.64	0.82	1.06		1.14	0.92	1.13
N6	0.53	0.97	1.12	0.91		0.84	1.45
N7	0.64	0.81	1.01	0.98	1.05		1.12
N8	0.42	0.83	0.85	0.77	1.17	0.69	

	HA-NA												
PB2	H1N1	H3N8	H4N6	H5N1	H5N2	H5N3	H6N1	H6N2	H6N5	H7N1	H7N3	H7N7	H9N2
H1N1		0.64	0.62	0.31	0.67	0.69	0.25	0.47	0.48	0.52	0.28	0.53	0.34
H3N8	0.40		0.65	0.16	0.55	0.50	0.16	0.46	0.33	0.30	0.14	0.31	0.18
H4N6	0.57	0.68		0.24	0.78	0.73	0.20	0.58	0.47	0.42	0.19	0.44	0.26
H5N1	0.03	0.03	0.03		0.03	0.03	0.03	0.03	0.03	0.03	0.03	0.03	0.03
H5N2	0.45	0.65	0.52	0.17		0.55	0.15	0.41	0.33	0.35	0.16	0.37	0.19
H5N3	0.48	0.57	0.60	0.20	0.59		0.12	0.41	0.34	0.35	0.15	0.36	0.18
H6N1	0.08	0.12	0.09	0.05	0.09	0.09		0.15	0.13	0.07	0.06	0.11	0.18
H6N2	0.21	0.35	0.27	0.10	0.32	0.24	0.15		0.30	0.17	0.08	0.20	0.12
H6N5	0.42	0.72	0.49	0.31	0.57	0.49	0.33	0.67		0.38	0.22	0.44	0.39
H7N1	0.48	0.49	0.41	0.21	0.52	0.52	0.15	0.35	0.37		0.28	0.41	0.21
H7N3	0.25	0.25	0.22	0.13	0.31	0.25	0.15	0.19	0.18	0.27		0.34	0.16
H7N7	0.48	0.54	0.44	0.26	0.56	0.50	0.24	0.45	0.42	0.42	0.26		0.33
H9N2	0.02	0.02	0.03	0.02	0.02	0.02	0.04	0.02	0.02	0.02	0.02	0.02	
PB1	H1N1	H3N8	H4N6	H5N1	H5N2	H5N3	H6N1	H6N2	H6N5	H7N1	H7N3	H7N7	H9N2
H1N1		0.78	0.80	0.27	0.75	0.87	0.47	0.85	0.61	0.58	0.24	0.73	0.24
H3N8	0.45		0.56	0.12	0.65	0.58	0.31	0.63	0.36	0.30	0.13	0.47	0.15
H4N6	0.61	0.85		0.20	0.80	0.81	0.47	0.85	0.48	0.39	0.19	0.66	0.20
H5N1	0.05	0.04	0.04		0.04	0.05	0.05	0.04	0.06	0.06	0.02	0.05	0.03

H5N2	0.53	0.68	0.67	0.15		0.72	0.37	0.76	0.38	0.31	0.16	0.58	0.22
H5N3	0.62	0.75	0.75	0.18	0.87		0.47	0.86	0.49	0.37	0.17	0.70	0.19
H6N1	0.13	0.15	0.20	0.07	0.25	0.20		0.31	0.13	0.11	0.07	0.21	0.08
H6N2	0.30	0.44	0.44	0.07	0.57	0.45	0.28		0.22	0.16	0.07	0.33	0.10
H6N5	0.48	0.59	0.57	0.22	0.55	0.53	0.41	0.57		0.39	0.22	0.49	0.24
H7N1	0.54	0.51	0.52	0.22	0.47	0.54	0.34	0.52	0.40		0.20	0.45	0.22
H7N3	0.12	0.14	0.13	0.38	0.15	0.13	0.15	0.15	0.10	0.12		0.15	0.17
H7N7	0.55	0.67	0.69	0.51	0.80	0.70	0.49	0.88	0.51	0.42	0.36		0.60
H9N2	0.05	0.05	0.05	0.03	0.05	0.05	0.05	0.05	0.04	0.05	0.04	0.05	
PA	H1N1	H3N8	H4N6	H5N1	H5N2	H5N3	H6N1	H6N2	H6N5	H7N1	H7N3	H7N7	H9N2
H1N1		0.47	0.48	0.27	0.51	0.52	0.37	0.37	0.48	0.39	0.26	0.42	0.36
H3N8	0.30		0.58	0.12	0.56	0.60	0.23	0.39	0.42	0.21	0.11	0.39	0.15
H4N6	0.33	0.72		0.25	0.86	0.76	0.40	0.51	0.74	0.26	0.15	0.42	0.26
H5N1	0.03	0.03	0.03		0.03	0.03	0.03	0.02	0.03	0.04	0.04	0.03	0.07
H5N2	0.27	0.51	0.66	0.22		0.68	0.32	0.40	0.65	0.22	0.12	0.30	0.18
H5N3	0.34	0.62	0.68	0.27	0.86		0.37	0.42	0.72	0.27	0.16	0.42	0.25
H6N1	0.16	0.20	0.21	0.06	0.17	0.19		0.16	0.16	0.13	0.09	0.15	0.12
H6N2	0.12	0.35	0.31	0.06	0.23	0.24	0.15		0.29	0.12	0.06	0.19	0.06
H6N5	0.27	0.58	0.54	0.24	0.48	0.44	0.45	0.88		0.22	0.16	0.30	0.19
H7N1	0.31	0.37	0.35	0.15	0.32	0.35	0.27	0.30	0.33		0.23	0.32	0.23
H7N3	0.16	0.17	0.18	0.13	0.17	0.17	0.15	0.17	0.15	0.19		0.15	0.21
H7N7	0.41	0.55	0.55	0.20	0.54	0.61	0.33	0.40	0.46	0.40	0.23		0.29
H9N2	0.04	0.04	0.05	0.03	0.05	0.05	0.06	0.05	0.05	0.04	0.03	0.04	
NP	H1N1	H3N8	H4N6	H5N1	H5N2	H5N3	H6N1	H6N2	H6N5	H7N1	H7N3	H7N7	H9N2
H1N1		0.51	0.63	0.31	0.59	0.49	0.35	0.48	0.52	0.37	0.24	0.54	0.38
H3N8	0.33		0.42	0.10	0.48	0.50	0.16	0.40	0.29	0.22	0.11	0.30	0.12
H4N6	0.43	0.53		0.16	0.56	0.50	0.24	0.56	0.53	0.27	0.14	0.41	0.21
H5N1	0.03	0.02	0.02		0.02	0.02	0.03	0.03	0.02	0.02	0.03	0.03	0.03
H5N2	0.40	0.44	0.45	0.22		0.50	0.14	0.33	0.27	0.21	0.12	0.33	0.29
H5N3	0.42	0.49	0.46	0.27	0.62		0.20	0.36	0.29	0.26	0.16	0.41	0.38
H6N1	0.08	0.11	0.09	0.04	0.08	0.08		0.17	0.14	0.09	0.08	0.08	0.05
H6N2	0.18	0.36	0.27	0.05	0.28	0.25	0.11		0.24	0.13	0.06	0.16	0.05
H6N5	0.29	0.42	0.42	0.12	0.30	0.28	0.25	0.69		0.21	0.15	0.28	0.13
H7N1	0.34	0.32	0.40	0.29	0.35	0.36	0.30	0.36	0.38		0.20	0.36	0.41
H7N3	0.20	0.21	0.20	0.16	0.20	0.19	0.27	0.24	0.18	0.19		0.20	0.22
H7N7	0.49	0.45	0.57	0.28	0.51	0.43	0.29	0.46	0.47	0.32	0.23		0.41
H9N2	0.04	0.04	0.04	0.03	0.03	0.04	0.03	0.03	0.04	0.03	0.03	0.03	
M	H1N1	H3N8	H4N6	H5N1	H5N2	H5N3	H6N1	H6N2	H6N5	H7N1	H7N3	H7N7	H9N2
H1N1		0.56	0.61	0.26	0.63	0.57	0.46	0.47	0.53	0.39	0.27	0.45	0.31
H3N8	0.33		0.61	0.10	0.52	0.57	0.23	0.42	0.45	0.20	0.10	0.24	0.18
H4N6	0.42	0.76		0.16	0.60	0.62	0.37	0.51	0.66	0.27	0.16	0.31	0.25
H5N1	0.03	0.02	0.02		0.02	0.03	0.02	0.03	0.03	0.03	0.03	0.03	0.03
H5N2	0.35	0.51	0.54	0.11		0.47	0.27	0.37	0.45	0.19	0.11	0.27	0.14
H5N3	0.47	0.71	0.72	0.16	0.62		0.32	0.48	0.52	0.30	0.16	0.35	0.23
H6N1	0.13	0.15	0.15	0.06	0.14	0.16		0.15	0.17	0.07	0.07	0.11	0.06
H6N2	0.17	0.36	0.30	0.05	0.30	0.29	0.14		0.21	0.09	0.06	0.13	0.08
H6N5	0.39	0.62	0.63	0.23	0.51	0.51	0.46	0.62		0.27	0.21	0.34	0.27
H7N1	0.36	0.35	0.35	0.18	0.39	0.36	0.25	0.27	0.28		0.22	0.31	0.20
H7N3	0.16	0.17	0.17	0.17	0.18	0.17	0.19	0.13	0.15	0.13		0.14	0.10
H7N7	0.35	0.38	0.39	0.23	0.42	0.39	0.33	0.34	0.37	0.31	0.24		0.24
H9N2	0.03	0.03	0.03	0.02	0.03	0.04	0.04	0.03	0.03	0.03	0.02	0.03	
NS	H1N1	H3N8	H4N6	H5N1	H5N2	H5N3	H6N1	H6N2	H6N5	H7N1	H7N3	H7N7	H9N2
H1N1		0.95	0.94	0.15	0.92	0.90	0.65	0.99	0.81	0.71	0.37	0.82	0.68
H3N8	0.53		0.88	0.05	0.97	0.82	0.19	0.92	0.61	0.41	0.14	0.49	0.18
H4N6	0.66	1.12		0.07	1.08	1.06	0.26	0.98	0.75	0.50	0.20	0.66	0.26
H5N1	0.17	0.12	0.10		0.11	0.10	0.17	0.11	0.12	0.13	0.09	0.12	0.17
H5N2	0.56	1.01	0.90	0.05		1.00	0.20	0.93	0.62	0.42	0.16	0.53	0.17
H5N3	0.67	1.07	1.10	0.09	1.11		0.25	0.96	0.74	0.48	0.19	0.62	0.23
H6N1	0.16	0.13	0.12	0.05	0.12	0.11		0.14	0.14	0.12	0.09	0.15	0.12
H6N2	0.41	0.71	0.62	0.05	0.76	0.62	0.17		0.50	0.30	0.12	0.37	0.15
H6N5	0.76	1.00	0.94	0.11	0.93	0.94	0.43	0.94		0.63	0.32	0.73	0.39
H7N1	0.53	0.62	0.66	0.11	0.65	0.66	0.29	0.58	0.53		0.27	0.54	0.31
H7N3	0.30	0.26	0.25	0.11	0.26	0.26	0.16	0.26	0.25	0.23		0.27	0.13
H7N7	0.74	0.86	0.85	0.14	0.87	0.77	0.47	0.82	0.76	0.67	0.37		0.47
H9N2	0.05	0.04	0.04	0.03	0.06	0.04	0.04	0.04	0.04	0.05	0.04	0.04	

Table S3 Partial correlation between reassortment and domestic proportion or dN/dS ratio

	R_{AB}	R_{BC}	R_{AC}	$R_{AB.C}$	$t_{AB.C}$	$P_{AB.C}$	$R_{AC.B}$	$t_{AC.B}$	$P_{AC.B}$
PB2	-0.67	0.58	-0.64	-0.47	2.95	0.01	-0.42	2.61	0.02
PB1	-0.69	0.46	-0.74	-0.59	3.87	0.003	-0.66	4.75	0.001
PA	-0.86	0.57	-0.68	-0.79	7.67	0.0001	-0.46	2.83	0.02
NP	-0.67	0.64	-0.64	-0.44	2.75	0.02	-0.37	2.35	0.04
M	-0.78	0.71	-0.62	-0.62	4.19	0.002	0.30	1.34	0.07
NS	-0.80	0.77	-0.73	-0.55	3.51	0.005	-0.29	1.97	0.07

A=inter-subtype reassortment rate;
B=dN/dS ratio;
C=host proportion (domestic);
$R_{AB.C}$ the partial correlation coefficient between reassortment rate and dN/dS independent of domestic proportion of 13 HA-NA subtypes;
$t_{AB.C}$: the t value of $R_{AB.C}$ with df =11;
$P_{AB.C}$: the two-tailed P value of the $t_{AB.C;}$
$R_{AC.B}$: the partial correlation coefficient between reassortment rate and domestic proportion independent of dN/dS of 13 HA-NA subtypes;
$t_{AC.B}$ the t value of $R_{AB.C}$ with df =11;
$P_{AC.B}$: the two-tailed P value of the $t_{AC.B.}$

Table S4 Number of selective sites of 13 subtypes and the correlation with inter-subtype reassortment rate in each internal segment.

		Number of negative selected sites						Number of sequences
Segment		PB2	PB1	PA	NP	M1	NS1	
	H1N1	53	21	29	23	8	12	24
	H3N8	214	145	183	112	32	33	22
	H4N6	195	115	119	69	17	16	12
	H5N1	337	521	327	246	252	34	234
	H5N2	324	295	209	163	55	46	24
	H5N3	129	87	153	79	42	22	14
	H6N1	336	305	289	183	62	47	66
	H6N2	484	458	374	239	83	75	140
	H6N5	38	38	13	20	1	0	12
	H7N1	4	29	7	1	2	6	40
	H7N3	25	129	26	16	6	10	33
	H7N7	40	66	70	27	11	7	12
	H9N2	492	491	423	266	97	72	114
R		-0.7	-0.77	-0.67	-0.79	-0.69	-0.55	
P-value		0.007	0.002	0.01	0.001	0.008	0.05	

Number of negative selected sites= the number of negative selected sites detected by SLAC for all six internal segments of each subtype.
Number of sequences=the number of sequences of each subtype being used in the analyses
R=correlation coefficient between the number of negatively selected sites and reassortment rate per subtype in each segment, with the P-value in the row below.

Table S5 Sequences for the discrete traits analysis

Information for subsampling data set A2 (n = 344, Table S1) of Eurasian AIV sequences used in this discrete traits analysis. Isolate name, subtype, host species, date of collection and the estimated decimal date are listed.

Isolate name	Subtype	Host species[a]	Date of collection	Decimal date
A/mallard/Marquenterre/Z237/1983	H1N1	ans	1983	1983.452
A/mallard/Bavaria/185-8/2008	H1N1	ans	22/09/2008	2008.723
A/goose/Italy/296426/2003	H1N1	ans	2003	2003.452
A/duck/Italy/281904/2006	H1N1	ans	2006	2006.452
A/duck/Italy/69238/2007	H1N1	ans	2007	2007.452
A/duck/Shimane/188/1999	H1N1	ans	1999	1999.452
A/commonteal/Netherlands/10/2000	H1N1	ans	23/11/2000	2000.893
A/Anasplatyrhynchos/Belgium/12827/2007	H3N8	ans	18/09/2007	2007.712
A/duck/Nanchang/1681/1992	H3N8	ans	01/12/1992	1992.915
A/duck/Beijing/40/04	H3N8	ans	2004	2004.452
A/duck/Beijing/61/05	H3N8	ans	2005	2005.452
A/mallard/Sanjiang/90/2007	H3N8	ans	2007	2007.452
A/mallard/CzechRepublic/13577-24K/2010	H3N8	ans	16/09/2010	2010.707
A/duck/Hokkaido/8/1980	H3N8	ans	1980	1980.452
A/mallard/Netherlands/3/2005	H3N8	ans	2005	2005.452
A/commoneider/Netherlands/1/2006	H3N8	ans	2006	2006.452
A/duck/Chabarovsk/1610/1972	H3N8	ans	1972	1972.452
A/gadwall/Altai/1326/2007	H3N8	ans	2007/09/	2007.704
A/mallard/Sweden/50/2002	H3N8	ans	14/11/2002	2002.868
A/duck/Ukraine/1/1963	H3N8	ans	12/12/1963	1963.945
A/duck/Vietnam/G119/2006	H3N8	ans	2006/11/	2006.871
A/duck/Zambia/04/2008	H3N8	ans	2008/06/	2008.452
A/goose/Zambia/06/2008	H3N8	ans	2008/07/	2008.534
A/turnstone/Netherlands/1/2007	H3N8	cha	2007	2007.452
A/chicken/Vietnam/G14/2008	H3N8	gal	2008/01/	2008.038
A/duck/Nanchang/4-165/2000	H4N6	ans	12/04/2000	2000.277
A/mallard/ZhaLong/88/2004	H4N6	ans	08/08/2004	2004.6
A/mallard/Yanchen/2005	H4N6	ans	2005/10/	2005.786
A/duck/CzechRepublic/1/1956	H4N6	ans	11/06/1956	1956.441
A/mallard/CzechRepublic/13579-84K/2010	H4N6	ans	13/09/2010	2010.699
A/duck/HongKong/365/1978	H4N6	ans	1978	1978.452
A/duck/Shiga/8/2004	H4N6	ans	2004	2004.452
A/mallard/Netherlands/1/1999	H4N6	ans	04/10/1999	1999.756
A/mallard/PT/35910-2/2006	H4N6	ans	30/10/2006	2006.827
A/mallard/Sweden/62/2003	H4N6	ans	05/09/2003	2003.677
A/goose/Zambia/07/2008	H4N6	ans	2008/09/	2008.704
A/dunlin/Sweden/1/2005	H4N6	cha	2005	2005.452
A/crestedeagle/Belgium/01/2004	H5N1	acc	2004	2004.452
A/mountainhawk-eagle/Kumamoto/1/07	H5N1	acc	2007	2007.452
A/duck/Cambodia/D14AL/2006	H5N1	ans	04/02/2006	2006.093
A/Goose/Guangdong/1/96	H5N1	ans	1996	1996.452

A/Duck/Anyang/AVL-1/2001	H5N1	ans	2001	2001.452
A/duck/Zhejiang/bj/2002	H5N1	ans	2002	2002.452
A/duck/China/E319-2/03	H5N1	ans	2003	2003.452
A/duck/Guangdong/173/04	H5N1	ans	2004	2004.452
A/domesticgreen-wingedteal/Hunan/79/2005	H5N1	ans	2005	2005.452
A/wildduck/Liaoning/8/2006	H5N1	ans	2006	2006.452
A/duck/Hunan/11/2007	H5N1	ans	2007	2007.452
A/duck/Hunan/3/2007	H5N1	ans	2007/10/	2007.786
A/duck/EasternChina/108/2008	H5N1	ans	15/12/2008	2008.953
A/duck/Zhejiang/2244/2011	H5N1	ans	2011/02/	2011.123
A/cygnusolor/Croatia/1/2005	H5N1	ans	2005	2005.452
A/duck/Egypt/2253-3/2006	H5N1	ans	2006	2006.452
A/duck/Egypt/D2br10/2007	H5N1	ans	2007/01/	2007.038
A/duck/Egypt/0871/2008	H5N1	ans	20/02/2008	2008.137
A/duck/France/080036/2008	H5N1	ans	2008/01/	2008.038
A/swan/Germany/R65/2006	H5N1	ans	2006/02/	2006.123
A/domesticduck/Germany/R1779/2007	H5N1	ans	2007	2007.452
A/goose/HongKong/485.3/2000	H5N1	ans	2000	2000.452
A/Duck/HongKong/380.5/2001	H5N1	ans	2001	2001.452
A/muteswan/Hungary/4571/2006	H5N1	ans	16/02/2006	2006.126
A/goose/Tripura/103596/2008	H5N1	ans	01/04/2008	2008.247
A/duck/EastJava/UT1046/2004	H5N1	ans	12/03/2004	2004.192
A/Cygnuscygnus/Iran/754/2006	H5N1	ans	2006	2006.452
A/mallard/Italy/3401/2005	H5N1	ans	2005	2005.452
A/mallard/Italy/835/2006	H5N1	ans	2006	2006.452
A/duck/Yokohama/aq10/2003	H5N1	ans	2003	2003.452
A/whooperswan/Akita/1/2008	H5N1	ans	2008	2008.452
A/mallard/Hokkaido/24/2009	H5N1	ans	2009	2009.452
A/duck/Fukushima/2/2011	H5N1	ans	2011/01/	2011.038
A/duck/Laos/25/2006	H5N1	ans	2006	2006.452
A/duck/Lao/981/2010	H5N1	ans	2010/03/	2010.2
A/whooperswan/Mongolia/244/2005	H5N1	ans	2005	2005.452
A/commongoldeneye/Mongolia/12/2006	H5N1	ans	2006/05/	2006.367
A/whooperswan/Mongolia/6/2009	H5N1	ans	2009/05/	2009.367
A/ruddyshelduck/Mongolia/X42/2009	H5N1	ans	2009/07/	2009.534
A/duck/Niger/914/2006	H5N1	ans	2006	2006.452
A/guineafowl/Nigeria/957-12/2006	H5N1	ans	2006	2006.452
A/goose/Krasnoozerskoye/627/2005	H5N1	ans	2005	2005.452
A/grebe/Tyva/Tyv06-1/2006	H5N1	ans	24/06/2006	2006.477
A/duck/Omsk/1822/2006	H5N1	ans	2006/07/	2006.534
A/Cygnuscygnus/Krasnodar/329/07	H5N1	ans	06/09/2007	2007.679
A/grebe/Tyva/3/2009	H5N1	ans	22/06/2009	2009.471
A/grebe/Tyva/2/2010	H5N1	ans	22/06/2010	2010.471
A/swan/Slovenia/760/2006	H5N1	ans	2006	2006.452
A/duck/Korea/ESD1/03	H5N1	ans	2003	2003.452

A/littlegrebe/Thailand/Phichit-01/2004	H5N1	ans	2004	2004.452
A/duck/Vietnam/7A/2004	H5N1	ans	2004	2004.452
A/duck/Vietnam/367/2005	H5N1	ans	2005	2005.452
A/duck/Vietnam/206/2005	H5N1	ans	07/12/2005	2005.932
A/muscovyduck/HaNam/07-47/2007	H5N1	ans	2007	2007.452
A/duck/Vietnam/G12/2008	H5N1	ans	2008/01/	2008.038
A/muscovyduck/Vietnam/OIE-559/2011	H5N1	ans	2011/06/	2011.452
A/commongull/Chany/P/2006	H5N1	cha	18/07/2006	2006.542
A/open-billstork/Thailand/VSMU-20-AYA/2004	H5N1	cic	2004	2004.452
A/open-billedstork/Nakhonsawan/BBD1821J/05	H5N1	cic	2005	2005.452
A/peregrinefalcon/HongKong/2142/2008	H5N1	fal	2008	2008.452
A/peregrinefalcon/Tochigi/15/2011	H5N1	fal	2011/02/	2011.123
A/sakerfalcon/Kuwait/0286/2007	H5N1	fal	2007	2007.452
A/falcon/SaudiArabia/D1795/2005	H5N1	fal	2005	2005.452
A/falcon/SaudiArabia/D1936/2007	H5N1	fal	2007	2007.452
A/chicken/Afghanistan/1573-47/2006	H5N1	gal	2006	2006.452
A/chicken/Bhutan/248009/2010	H5N1	gal	20/02/2010	2010.137
A/chicken/Cambodia/TKCMB5T/2010	H5N1	gal	28/01/2010	2010.074
A/chicken/Hubei/wj/1997	H5N1	gal	1997	1997.452
A/chicken/Hebei/718/2001	H5N1	gal	2001	2001.452
A/chicken/Hebei/108/02	H5N1	gal	2002	2002.452
A/chicken/Hubei/wo/2003	H5N1	gal	2003	2003.452
A/chicken/Jiangxi/25/2004	H5N1	gal	2004	2004.452
A/chicken/Guangdong/1/2005	H5N1	gal	2005	2005.452
A/chicken/Liaoning/A-11/2006	H5N1	gal	2006	2006.452
A/chicken/Liaoning/A-1/2007	H5N1	gal	2007	2007.452
A/chicken/Jiangsu/18/2008	H5N1	gal	2008	2008.452
A/chicken/Hunan/1/2009	H5N1	gal	2009	2009.452
A/chicken/IvoryCoast/1787-35/2006	H5N1	gal	2006	2006.452
A/turkey/IvoryCoast/4372-2/2006	H5N1	gal	2006/12/	2006.953
A/turkey/Egypt/2253-2/2006	H5N1	gal	2006	2006.452
A/chicken/Egypt/CL6/2007	H5N1	gal	2007/03/	2007.2
A/chicken/Egypt/0836/2008	H5N1	gal	15/01/2008	2008.038
A/chicken/Egypt/0891/2008	H5N1	gal	26/09/2008	2008.734
A/chicken/Germany/R3234/2007	H5N1	gal	2007	2007.452
A/Ck/HK/YU22/2002	H5N1	gal	2002	2002.452
A/chicken/India/NIV33487/06	H5N1	gal	2006	2006.452
A/chicken/India/WB-NIV2653/2008	H5N1	gal	21/01/2008	2008.055
A/chicken/WestBengal/170564/2009	H5N1	gal	09/03/2009	2009.184
A/chicken/WestBengal/239020/2010	H5N1	gal	12/01/2010	2010.03
A/quail/Yogjakarta/UT1023/2004	H5N1	gal	15/01/2004	2004.038
A/chicken/CentralJava/UT3091/2005	H5N1	gal	2005	2005.452
A/chicken/SouthKalimantan/UT6028/2006	H5N1	gal	2006/11/	2006.871
A/chicken/Oita/8/2004	H5N1	gal	2004	2004.452
A/chicken/Kuwait/KISR8/2007	H5N1	gal	2007	2007.452

A/chicken/Laos/P0130/2007	H5N1	gal	2007	2007.452
A/chicken/Lao/LH1/2010	H5N1	gal	2010/05/	2010.367
A/chicken/Nigeria/1047-62/2006	H5N1	gal	2006	2006.452
A/chicken/Nigeria/OG11/2007	H5N1	gal	2007	2007.452
A/chicken/Rawalakot/NARC2441A/2006	H5N1	gal	2006	2006.452
A/turkey/Poland/R3249/2007	H5N1	gal	2007	2007.452
A/chicken/Kurgan/3/2005	H5N1	gal	2005	2005.452
A/chicken/Reshoty/02/2006	H5N1	gal	2006/06/	2006.452
A/chicken/Russia/Krasnodar/2/2007	H5N1	gal	2007	2007.452
A/chicken/Krasnodar/300/07	H5N1	gal	05/09/2007	2007.677
A/turkey/SaudiArabia/6732-6/2007	H5N1	gal	2007	2007.452
A/chicken/Sudan/1784-10/2006	H5N1	gal	2006	2006.452
A/chicken/KohnKaen/NIAH330/2004	H5N1	gal	2004	2004.452
A/quail/NakhonPathom/NIAH7562/2005	H5N1	gal	2005	2005.452
A/chicken/Phichit/NIAH606988/2006	H5N1	gal	2006	2006.452
A/chicken/Sukhothai/NIAH114843/2008	H5N1	gal	2008	2008.452
A/chicken/Crimea/08/2005	H5N1	gal	2005	2005.452
A/chicken/Scotland/1959	H5N1	gal	1959	1959.452
A/chicken/Vietnam/921/2004	H5N1	gal	2004	2004.452
A/chicken/LangSon/200/2005	H5N1	gal	2005	2005.452
A/chicken/Vietnam/209/2005	H5N1	gal	08/12/2005	2005.934
A/chicken/NamDinh/07-32/2007	H5N1	gal	2007	2007.452
A/piedmagpie/Liaoning/7/2006	H5N1	pas	2006	2006.452
A/magpierobin/HongKong/1897/2008	H5N1	pas	2008	2008.452
A/crow/Assam/142119/2008	H5N1	pas	2008/12/	2008.953
A/crow/Osaka/102/2004	H5N1	pas	2004	2004.452
A/pigeon/Laos/P0022/2007	H5N1	pas	2007	2007.452
A/pigeon/Rostov-on-Don/6/2007	H5N1	pas	14/12/2007	2007.951
A/pigeon/Thailand/VSMU-7-NPT/2004	H5N1	pas	2004	2004.452
A/pigeon/Thailand/VSMU-11-KRI/2005	H5N1	pas	2005	2005.452
A/egret/HongKong/757.2/2003	H5N1	pel	2003	2003.452
A/greyheron/HongKong/3088/2007	H5N1	pel	2007	2007.452
A/greyheron/HongKong/1046/2008	H5N1	pel	2008	2008.452
A/ostrich/Suzhou/097/2003	H5N1	str	16/04/2003	2003.288
A/ostrich/Nigeria/1047-25/2006	H5N1	str	2006	2006.452
A/garganey/SanJiang/160/2006	H5N2	ans	2006	2006.452
A/duck/Denmark/65047/04	H5N2	ans	2004	2004.452
A/duck/France/080032/2008	H5N2	ans	2008/01/	2008.038
A/duck/Potsdam/1402-6/1986	H5N2	ans	1986	1986.452
A/duck/HongKong/342/78	H5N2	ans	1978	1978.452
A/teal/Italy/3931-38/2005	H5N2	ans	2005	2005.452
A/mallard/Italy/4223-2/2006	H5N2	ans	11/11/2006	2006.86
A/northernpintail/Akita/714/2006	H5N2	ans	2006/12/	2006.953
A/duck/Malaysia/F118-08-04/2004	H5N2	ans	2004	2004.452
A/mallard/Netherlands/3/1999	H5N2	ans	09/10/1999	1999.77
A/duck/Primorie/2621/2001	H5N2	ans	24/08/2001	2001.644

A/gadwall/Altai/1202/2007	H5N2	ans	2007/09/	2007.704
A/mallard/Sweden/74/2003	H5N2	ans	2003	2003.452
A/turkey/Italy/1980	H5N2	gal	1980	1980.452
A/poultry/Italy/382/1997	H5N2	gal	1997	1997.452
A/turkey/Italy/1258/2005	H5N2	gal	2005	2005.452
A/duck/France/02166/2002	H5N3	ans	2002	2002.452
A/duck/France/06436/2006	H5N3	ans	21/03/2006	2006.216
A/mallard/France/061054/2006	H5N3	ans	07/11/2006	2006.849
A/duck/HongKong/205/1977	H5N3	ans	1977	1977.452
A/goose/HongKong/23/1978	H5N3	ans	1978	1978.452
A/duck/Italy/775/2004	H5N3	ans	2004	2004.452
A/teal/Italy/3812/2005	H5N3	ans	2005	2005.452
A/mallard/Netherlands/65/2006	H5N3	ans	2006	2006.452
A/duck/Altai/1285/1991	H5N3	ans	15/08/1991	1991.619
A/duck/Primorie/2633/2001	H5N3	ans	24/08/2001	2001.644
A/spot-billedduck/Korea/KNUSYG06/2006	H5N3	ans	2006	2006.452
A/tern/SouthAfrica/1961	H5N3	cha	1961	1961.452
A/mallard/Jiangxi/7787/2003	H6N1	ans	2003	2003.452
A/duck/Shantou/1275/2004	H6N1	ans	2004	2004.452
A/mallard/Jiangxi/13228/2005	H6N1	ans	2005	2005.452
A/duck/EasternChina/1/2008	H6N1	ans	2008/11/	2008.871
A/duck/HongKong/d73/1976	H6N1	ans	1976	1976.452
A/Teal/HongKong/W312/97	H6N1	ans	1997	1997.452
A/greylaggoose/Netherlands/4/1999	H6N1	ans	01/03/1999	1999.162
A/mallard/Sweden/81/2002	H6N1	ans	03/12/2002	2002.921
A/mallard/Sweden/30/2005	H6N1	ans	2005	2005.452
A/duck/Taiwan/0526/72	H6N1	ans	1972	1972.452
A/duck/Taiwan/WB29/99	H6N1	ans	1999	1999.452
A/duck/Taiwan/29-3/00	H6N1	ans	2000	2000.452
A/duck/Taiwan/WB239/03	H6N1	ans	2003	2003.452
A/chicken/HongKong/17/1977	H6N1	gal	1977	1977.452
A/quail/HongKong/1721-30/99	H6N1	gal	1999	1999.452
A/quail/HongKong/SF595/00	H6N1	gal	2000	2000.452
A/pheasant/HongKong/SSP44/2002	H6N1	gal	2002	2002.452
A/chukkar/HongKong/SF126/2003	H6N1	gal	2003	2003.452
A/chicken/Taiwan/0824/97	H6N1	gal	1997	1997.452
A/chicken/Taiwan/na3/98	H6N1	gal	1998	1998.452
A/chicken/Taiwan/0705/99	H6N1	gal	1999	1999.452
A/chicken/Taiwan/SP1/00	H6N1	gal	2000	2000.452
A/chicken/Taiwan/1205/01	H6N1	gal	2001	2001.452
A/chicken/Taiwan/0208/02	H6N1	gal	2002	2002.452
A/chicken/Taiwan/1203/03	H6N1	gal	2003	2003.452
A/chicken/Taiwan/ch1006/04	H6N1	gal	2004	2004.452
A/chicken/Taiwan/A342/05	H6N1	gal	2005	2005.452
A/pigeon/HongKong/WF47/2003	H6N1	pas	2003	2003.452

A/partridge/Taiwan/LU1/99	H6N1	pas	1999	1999.452
A/wildduck/Shantou/1651/2000	H6N2	ans	2000	2000.452
A/duck/Shantou/2444/2001	H6N2	ans	2001	2001.452
A/duck/Shantou/2471/2002	H6N2	ans	2002	2002.452
A/mallard/Jiangxi/7376/2003	H6N2	ans	2003	2003.452
A/mallard/Jiangxi/8264/2004	H6N2	ans	2004	2004.452
A/duck/Shantou/7568/2005	H6N2	ans	2005	2005.452
A/mallard/HeiLongjiang/131/2006	H6N2	ans	2006	2006.452
A/duck/EasternChina/40/2007	H6N2	ans	2007/05/	2007.367
A/duck/EasternChina/46/2008	H6N2	ans	2008/04/	2008.285
A/duck/EasternChina/2/2008	H6N2	ans	2008/12/	2008.953
A/mallard/CzechRepublic/15902-17K/2009	H6N2	ans	30/09/2009	2009.745
A/duck/HongKong/d134/1977	H6N2	ans	1977	1977.452
A/whitefrontedgoose/Netherlands/2/1999	H6N2	ans	02/03/1999	1999.164
A/barnaclegoose/Netherlands/1/2005	H6N2	ans	2005	2005.452
A/mallard/Netherlands/71/2006	H6N2	ans	2006	2006.452
A/aquaticbird/Korea/CN17/2009	H6N2	ans	2009/11/	2009.871
A/mallard/Sweden/52/2003	H6N2	ans	30/10/2003	2003.827
A/duck/Kingmen/E322/04	H6N2	ans	2004	2004.452
A/Muscovyduck/Vietnam/G33/2007	H6N2	ans	2007/09/	2007.704
A/duck/Zambia/03/2008	H6N2	ans	2008/06/	2008.452
A/duck/Zambia/10/2009	H6N2	ans	2009/09/	2009.704
A/gull/Moscow/3100/2006	H6N2	cha	2006/10/	2006.786
A/chicken/Hunan/989/2005	H6N2	gal	2005	2005.452
A/chicken/EasternChina/42/2007	H6N2	gal	2007/06/	2007.452
A/duck/Hunan/5613/2003	H6N5	ans	2003	2003.452
A/mallard/Jiangxi/8346/2004	H6N5	ans	2004	2004.452
A/duck/Hunan/748/2005	H6N5	ans	2005	2005.452
A/duck/Yangzhou/013/2008	H6N5	ans	15/12/2008	2008.953
A/mallard/Netherlands/11/2007	H6N5	ans	2007	2007.452
A/aquaticbird/Korea/W69/2005	H6N5	ans	2005/11/	2005.871
A/aquaticbird/Korea/CN5/2009	H6N5	ans	2009/11/	2009.871
A/duck/Taiwan/WB459/04	H6N5	ans	2004	2004.452
A/duck/Nanchang/1904/1992	H7N1	ans	1992	1992.452
A/duck/Denmark/53-147-8/2008	H7N1	ans	25/04/2008	2008.312
A/guineafowl/Italy/155/2000	H7N1	ans	11/01/2000	2000.027
A/duck/Mongolia/47/2001	H7N1	ans	2001	2001.452
A/duck/Mongolia/867/2002	H7N1	ans	2002	2002.452
A/mallard/Netherlands/22/2007	H7N1	ans	2007	2007.452
A/chicken/Italy/1391/1999	H7N1	gal	16/04/1999	1999.288
A/chicken/Italy/2335/2000	H7N1	gal	31/03/2000	2000.244
A/turkey/Italy/1351/2001	H7N1	gal	15/02/2001	2001.123
A/ostrich/Italy/984/00	H7N1	str	2000	2000.452
A/mallard/Netherlands/12/2000	H7N3	ans	2000	2000.452
A/tuftedduck/PT/13771/2006	H7N3	ans	22/03/2006	2006.219
A/turkey/Italy/8534/2002	H7N3	gal	2002	2002.452

A/duck/Korea/BC10/2007	H7N3	ans	2007/03/	2007.216
A/duck/Shimane/137/2006	H7N3	ans	2006	2006.452
A/chicken/Pakistan/34669/1995	H7N3	gal	1995	1995.452
A/chicken/Pakistan/c1998/1998	H7N3	gal	1998	1998.452
A/chicken/Chakwal/NARC-35/2001	H7N3	gal	2001/05/	2001.367
A/chicken/Rawalpindi/NARC68/2002	H7N3	gal	2002	2002.452
A/chicken/Chakwal/NARC-148/2004	H7N3	gal	12/05/2004	2004.359
A/swan/CzechRepublic/5416/2011	H7N7	ans	06/04/2011	2011.26
A/mallard/Netherlands/9/2005	H7N7	ans	2005	2005.452
A/swan/Slovenia/53/2009	H7N7	ans	20/01/2009	2009.052
A/duck/Tsukuba/664/2007	H7N7	ans	2007/12/	2007.953
A/mallard/Sweden/95/2005	H7N7	ans	2005	2005.452
A/chicken/Germany/R28/03	H7N7	gal	2003	2003.452
A/chicken/Netherlands/1/03	H7N7	gal	2003	2003.452
A/duck/Korea/JSM/2010	H7N7	ans	2010/06/	2010.452
A/duck/Nanjing/2/97	H9N2	ans	1997	1997.452
A/wildduck/Nanchang/2-0480/2000	H9N2	ans	17/02/2000	2000.129
A/duck/Hubei/W1/2004	H9N2	ans	2004	2004.452
A/duck/Guangxi/51/2005	H9N2	ans	2005	2005.452
A/duck/Fujian/FQ107/2007	H9N2	ans	2007/12/	2007.953
A/duck/Tibet/S2/2009	H9N2	ans	10/09/2009	2009.69
A/mallard/France/090360/2009	H9N2	ans	2009/09/	2009.704
A/duck/HongKong/702/1979-quailadapted	H9N2	ans	1979	1979.452
A/duck/HongKong/784/1979	H9N2	ans	10/10/1979	1979.773
A/duck/Hokkaido/49/98	H9N2	ans	1998	1998.452
A/duck/Hokkaido/9/99	H9N2	ans	1999	1999.452
A/duck/Malaysia/2001	H9N2	ans	2001	2001.452
A/Eurasianwigeon/Netherlands/3/2005	H9N2	ans	2005	2005.452
A/gadwall/Netherlands/1/2006	H9N2	ans	2006	2006.452
A/Bewicksswan/Netherlands/5/2007	H9N2	ans	2007	2007.452
A/duck/Korea/KNUDPJ09/2009	H9N2	ans	2009/05/	2009.367
A/chicken/Shandong/7/96	H9N2	gal	1996	1996.452
A/chicken/Shenzhen/9/97	H9N2	gal	1997	1997.452
A/chicken/Beijing/8/98	H9N2	gal	1998	1998.452
A/chicken/Ningxia/4/99	H9N2	gal	1999	1999.452
A/quail/Nanchang/2-0460/2000	H9N2	gal	17/02/2000	2000.129
A/chicken/China/Guangxi14/2000	H9N2	gal	13/11/2000	2000.866
A/chicken/Henan/43/02	H9N2	gal	2002	2002.452
A/chicken/Guangxi/37/2005	H9N2	gal	2005	2005.452
A/chicken/Hebei/L1/2006	H9N2	gal	2006	2006.452
A/chicken/Hubei/C1/2007	H9N2	gal	2007	2007.452
A/chicken/Henan/1.2/2008	H9N2	gal	10/01/2008	2008.025
A/chicken/Hebei/Y2/2009	H9N2	gal	2009/03/	2009.2
A/chicken/Tibet/S1/2009	H9N2	gal	10/09/2009	2009.69
A/chicken/China/AH-10-01/2010	H9N2	gal	02/12/2010	2010.918
A/chicken/Guangdong/ZCY/2011	H9N2	gal	2011/07/	2011.534

A/chicken/Uchal/8286/2006	H9N2	gal	25/02/2006	2006.151
A/chicken/Israel/90658/2000	H9N2	gal	2000	2000.452
A/turkey/Israel/810/2001	H9N2	gal	2001	2001.452
A/turkey/Israel/619/2002	H9N2	gal	2002	2002.452
A/chicken/Israel/1475/2003	H9N2	gal	2003	2003.452
A/chicken/Israel/1966/2004	H9N2	gal	2004	2004.452
A/chicken/Israel/554/2005	H9N2	gal	2005	2005.452
A/chicken/Israel/178/2006	H9N2	gal	2006	2006.452
A/chicken/Israel/375/2007	H9N2	gal	06/03/2007	2007.175
A/chicken/Israel/310/2008	H9N2	gal	27/06/2008	2008.485
A/chicken/Israel/184/2009	H9N2	gal	15/02/2009	2009.123
A/chicken/Osaka/aq48/97	H9N2	gal	1997	1997.452
A/chicken/Osaka/aq19/2001	H9N2	gal	2001	2001.452
A/chicken/Yokohama/aq45/2002	H9N2	gal	2002	2002.452
A/quail/Lebanon/272/2010	H9N2	gal	2010/08/	2010.619
A/chicken/Pakistan/2/99	H9N2	gal	1999	1999.452
A/chicken/Pakistan/UDL-01/2005	H9N2	gal	18/05/2005	2005.375
A/chicken/Pakistan/UDL-03/2005	H9N2	gal	21/12/2005	2005.97
A/chicken/Pakistan/UDL-01/2007	H9N2	gal	07/06/2007	2007.43
A/chicken/Pakistan/UDL-03/2008	H9N2	gal	04/03/2008	2008.17
A/Korea/KBNP-0028/2000	H9N2	gal	2000	2000.452
A/chicken/Korea/S21/2004	H9N2	gal	2004	2004.452
A/chicken/Korea/GH2/2007	H9N2	gal	24/11/2007	2007.896
A/chicken/Korea/KNUSWR09/2009	H9N2	gal	2009/04/	2009.285
A/chicken/Dubai/338/2001	H9N2	gal	2001	2001.452
A/chicken/Emirates/R66/2002	H9N2	gal	2002	2002.452
A/pigeon/Nanchang/2-0461/2000	H9N2	pas	17/02/2000	2000.129
A/black-billedmagpie/Guangxi/31/2005	H9N2	pas	2005/05/	2005.367
A/parakeet/Chiba/1/97	H9N2	psi	1997	1997.452
A/parakeet/Narita/92A/98	H9N2	psi	1998	1998.452

[a]: The abbreviations of bird orders being used in this study:
acc: Accipitriformes
ans: Anseriformes
cha: Charadriiformes
cic: Ciconiiformes
cor Coraciiformes
col Columbiformes
cuc Cuculiformes
gal: Galliformes
gru: Gruiformes
Fal: Falconiformes
pas: Passeriformes
pel Pelecaniforms
pic Piciformes
pro: Procellariiformes
psi Psittaciformes
rhe: Rheiformes

str: Struthioniformes
